# R&D mode and coordination of green products in sustainable supply chain considering power structures

**DOI:** 10.1371/journal.pone.0291351

**Published:** 2023-11-02

**Authors:** Tong Liu, Qinghua Feng

**Affiliations:** School of Information, Xi’an University of Finance and Economics, Xi’an, China; Xuzhou University of Technology, CHINA

## Abstract

Green product R&D has a significant impact on the sustainable development of the economy and environment, and green product R&D can be carried out by manufacturers, retailers, third-party companies, and enterprise alliances. The decision-making order in the supply chain depends on the power structures, which can affect the choice of the supply chain decision-making. To study the optimal choice of green product R&D mode in different power structures and the influence of power structure on product price, market demand, green level, and enterprise profits, This paper compares five modes including green product R&D by manufacturers, green product R&D by retailers, green product R&D outsourced by manufacturers to third-party companies, green product R&D outsourced by retailers to third-party companies and green product R&D by manufacturers and retailers in the three power structures of manufacturers as core enterprises, retailers as core enterprises, and equal power between manufacturers and retailers to study the selection strategy of green product R&D modes in the sustainable supply chain. The conclusion provides a strategic reference for the selection of green product R&D mode in different power structures. The findings indicate that when manufacturers are core enterprises, retailers’ green product R&D is better than that of manufacturers. When retailers are core enterprises, manufacturers’ green product R&D is better than that of retailers. In the same power structure, manufacturers’ green product R&D is better than outsourcing to third-party companies, retailers’ green product R&D is better than outsourcing to third-party companies, and manufacturers and retailers jointly conduct green product R&D better than manufacturers or retailers alone. When manufacturers and retailers have equal power, the market demand and the product green level are the highest, and the retail price is the lowest. When manufacturers are core enterprises, manufacturers’ profits are the highest. When retailers are core enterprises, retailers’ profits are the highest. Finally, a two-part pricing contract is used to coordinate the optimal selection strategies.

## 1. Introduction

With the rapid development of the global economy, the earth’s resources are becoming scarce, environmental pollution is becoming increasingly severe. As a result, environmental protection has gained the attention of countries in the world. In addition, people’s quality of life has improved, and their awareness of ecological environmental protection has increased, leading consumers to seek out healthy and friendly products. Enterprises and scholars have become highly concerned with the research of sustainable supply chains [1–3]. Achieving green sustainable development has also attracted the attention of governments around the world, and a series of laws and regulations have been issued, which have played a positive role in promoting manufacturers and retailers to achieve green product R&D [4]. Green product R&D not only promotes market demand but also improves the competitive advantage of enterprises.

Many enterprises are focusing on green product R&D to combat the global resource shortage and environmental deterioration. For example, Lenovo, as a manufacturer, has reduced its carbon emissions by 35% through technological research and innovation of low-temperature welding manufacturing processes. As a retailer, Wal-Mart develops green packaging and minimizes unnecessary packaging. In addition, some companies outsource the R&D and innovation of green products to professional third-party companies due to their technology and resource limitations. BMW, for instance, has outsourced the R&D of environmentally-friendly batteries for electric vehicles to CATL. Goodyear, the world’s leading tire company, has been commissioned by Buick to develop a new generation of green tires with low-rolling resistance. Additionally, Goodyear plans to cooperate with PATAC to develop another generation of low-rolling resistance tires. In 2017, PATAC worked with Axalta, the world’s leading supplier of liquid and powder coatings, to create an environmentally friendly and aesthetically pleasing waterborne coating for new energy vehicles.

It is evident that various enterprises involved in sustainable supply chains can engage in green product R&D. These enterprises can be manufacturers, retailers, enterprise alliances between manufacturers and retailers, and professional third-party companies. This paper categorizes the different green product R&D modes based on the type of enterprise involved and analyzes which one is the best. Additionally, the power structure of the supply chain, which could be dominated by large manufacturers such as IBM, large retailers such as Wal-Mart, or have an equal power between manufacturers and retailers, plays a significant role in the decision-making order of supply chain enterprises. Previous studies have shown that the power structure affects the decision-making results of the supply chain and the profits of enterprises, which then affects the optimal choice of green product R&D mode.

The purpose of this study is to compare and analyze five different green product R&D modes in different power structures of sustainable supply chains by establishing game models. The five modes include green product R&D by manufacturers, green product R&D by retailers, green product R&D by manufacturers outsourcing to third-party companies, green product R&D by retailers outsourcing to third-party companies, and green product R&D by manufacturers and retailers. The aim is to determine the optimal green product R&D mode in different power structures and to assess the impact of power structures on green product price, green level, demand, enterprise profits, and optimal decision selection. Finally, supply chain coordination is realized through two-pricing contracts for the optimal green product R&D mode.

Therefore, this paper raises the following questions: (1) Which R&D mode of green products is the best in different power structures? (2) Is it homemade or outsourced for manufacturers or retailers to carry out green product R&D? Do manufacturers and retailers independently or jointly conduct green product R&D? (3) How does the power structure affect the market demand, retail price, and green level of products and the choice of the optimal green R&D mode? (4) How to coordinate the supply chain for the optimal strategy of green product R&D mode?

To further explore the above research questions and fill in the corresponding research gaps, this paper mainly studies the optimal R&D modes of green products in the three power structures: manufacturers as the core enterprise, retailers as the core enterprise, and equal power between manufacturers and retailers. Finally, the optimal modes are coordinated through two-part pricing contracts.

The main contributions of this paper are as follows:

1. This paper compares and analyzes five green product R&D modes in different power structures. The modes include manufacturers conduct green product R&D, retailers conduct green product R&D, manufacturers and retailers jointly conduct green product R&D, manufacturers outsource green product R&D to third-party companies and retailers outsource green product R&D to third-party companies, and obtains the optimal green product R&D modes.
2. This paper examines the impact of power structures on market demand, product price, product green level, and enterprise profits. It is concluded that when manufacturers and retailers have equal power, the market demand and green level of products are the highest, and the retail price of products is the lowest. When manufacturers are core enterprises, the profits of manufacturers are the highest. When retailers are core enterprises, the profits of retailers are the highest.
3. This study indicates that in the same power structure, manufacturers’ green product R&D is better than manufacturers outsourcing to third-party companies. Retailers’ green product R&D is better than retailers outsourcing to third-party companies. Manufacturers and retailers jointly conduct green product R&D better than manufacturers or retailers independently.

The rest of this paper is structured as follows. The second part is a literature review, the third part introduces the assumptions and symbols of the game model, the fourth part is the game models of green product R&D modes in different power structures, the fifth part analyzes the equilibrium results of five green product R&D modes in different power structures, the sixth part is supply chain coordination of the two-part pricing contract, the seventh part is the numerical simulation of the conclusion, the eighth part summarizes the research results and puts forward the future research direction.

## 2. Literature review

This paper is closely related to power structures, green innovation and green product R&D, and supply chain coordination. This section will sort out and review relevant literatures, and point out the differences between the existing research and the study in this paper.

### 2.1. Power structures of the supply chain

In terms of the study of the power structure of the supply chain, Wang et al. [5] studied a two-level supply chain consisting of a manufacturer and two competing retailers. By constructing a decentralized decision-making model of three vertical power structures and two horizontal power structures of retailers, and concluded the influence of power structure and consumer environmental awareness on the green level and profit of supply chain. Liu et al. [6] constructed game models of manufacturer-led, retailer-led, producer-led, and retailer-led with wholesale price contracts. The research showed that no matter what kind of power structure, the manufacturer’s wholesale price with the revenue-sharing contract is higher than the manufacturer’s wholesale price with the wholesale price contract. When the retailer was dominated, the retailer’s sales price with the revenue-sharing contract is higher than the wholesale price. Su et al. [7] analyzed the government subsidy strategies of manufacturer-dominated and retailer-dominated. The results showed that the wholesale price dominated by the manufacturer is higher than that dominated by the retailer. Zand et al. [8] considered a supply chain consisting of one manufacturer and two retailers, constructed game models of cooperation and non-cooperation between the manufacturer and the government when the retailer is a dominant enterprise, and discussed the influence of the dominance of retailers on the level of cooperation between the manufacturers and the government. Xu et al. [9] explored the difference in consumers’ perception of the green level of products in different channels, and constructed game models of manufacturer-dominated, retailer-dominated and equal power between manufacturers and retailers, and found that the equal power structure of manufacturers and retailers can improve the green level of products and the profitability of supply chain. The green level of products and the profit of manufacturers in the two-channel structure are higher than those in the single-channel structure. TMR et al. [10] discussed the dual-channel supply chain composed of physical retailers and online retailers, constructed a game model of physical store dominance, electronic store dominance, and equal power between physical stores and electronic stores, and concluded that the competition between physical stores and electronic stores with the same power would make their profits lower. Yang et al. [11] explored government intervention and consumer green preference, and constructed game models in three power structures: manufacturer-dominated, retailer-dominated, and Nash game, and found that the manufacturer Stackelberg is sub-optimal, while the retailer Stackelberg is superior to others. Liu et al. [12] analyzed the influence of five power structures on supply chain operation, and the results showed that government subsidies had a positive impact on the energy-saving level of green products, the financial performance of enterprises, and the profit of the supply chain. Liu et al. [13] considered consumers’ preference for CSR products and studied the decision-making of green supply chains dominated by manufacturers, retailers, and equal power between manufacturers and retailers. It is found that the higher consumer preference, the higher product sales and corporate social responsibility. Xue K et al. [14] explored the strategic choice of green product design considering government subsidies in different power structures. It is found that the product green level, market demand, and profits of enterprises are higher when the retailer is the core enterprise. Huang et al. [15] discussed the influence of corporate social responsibility, and power structures in green supply chains on green marketing decision-making and found that corporate social responsibility is conducive to improving product green level and increasing corporate profits. When the other party assumes social responsibility, the green level and profit of the supply chain are higher. Xia et al. [16] studied the impact of cross-shareholding on prices, carbon emission reduction, and corporate profits in different power structures, and found that when the proportion of cross-shareholding meets certain conditions, the profits of both manufacturers and retailers will increase. Manufacturers’ and retailers’ carbon reduction rates are influenced by their share ratio and power structures. Agi et al. [17] analyzed the pricing strategies of ordinary products and green products in different power structures and gave the conditions under which green products are more profitable than ordinary products. The study also showed that a manufacturer-led supply chain is more willing to produce green products than a retailer-led supply chain. Jena et al. [18] constructed a closed-loop supply chain model for omnichannel retailing in different channel power structures, studied the impact of testing in-store and buying online on supply chain profits, and adopted two-part tariff contracts for supply chain coordination. It is found that manufacturers and remanufacturers are more profitable when the manufacturer is dominated, and retailers are more profitable when the retailer is dominated and the power is equal. Zhang et al. [19] investigated the production and emission reduction strategies of manufacturers and emission allowances of the government in different power structures and found that if manufacturers adopt green technology strategies, the power structure of the supply chain will not affect the social welfare, and the social welfare of the government is the largest in the structure of equal power. Zhao et al. [20] discussed the optimal decision-making and government subsidy strategies of photovoltaic supply chain enterprises in different power structure and found that reasonable government subsidies can provide the profits and social welfare of enterprises in the supply chain. Different power structures have significant effects on optimal government subsidies and optimal decision-making of supply chain enterprises but do not affect social welfare. Zhang et al. [21] compared the pricing and green advertising decisions of four kinds of green advertising investment of the two companies in different power structures. The research found that when the two companies did not invest in green advertising, the profit was lowest, while when they invested in green advertising, the profit was highest. When only one invests in green advertising, Stackelberg followers benefit from investing in green advertising. Li et al. [22] studied the influence of two kinds of government subsidies based on fixed green technology investment cost subsidy (FC) and emission reduction subsidy (ER) on green decision-making in three power structures and found that in the same amount of subsidies, manufacturers with FC subsidy would have higher profits and fewer emissions while retailers with ER subsidy would have higher profits.

The above literature studies the influence of power structures on green supply chain selection decisions from the perspectives of consumers’ environmental awareness, coordination contracts, government subsidies, enterprise cooperation, advertising, green financing, channel structures and government intervention. This paper studies the influence of power structure on the strategic choice of sustainable supply chain from the perspective of green product R&D modes.

### 2.2 Green innovation and green product R&D

In the green innovation and green product R&D of supply chain, Chen et al. [23] considered a two-level supply chain composed of manufacturers and retailers, and constructed game models of manufacturer-dominated, retailer-dominated, and manufacturer and retailer with equal power structure. The research found that green product R&D cooperation had a positive impact on the environment, consumer surplus and social welfare, and technology spillover and bargaining power affected economic performance. Ge et al. [24] studied the R&D cooperation behavior of enterprises in supply chain. The research showed that only when the contribution level of both parties was equivalent, both parties achieved a win-win. Yang et al. [25] analyzed green R&D cooperation in supply chain and used revenue-sharing contracts to coordinate the supply chain. The results showed that Pareto improvement could be achieved only when manufacturers effectively encouraged retailers to cooperate. Dai et al. [26] constructed and compared a game model of R&D and non-R&D cooperation considering government subsidies and consumer awareness. The results showed that a cost-sharing contract brought more profits to members than a non-cooperative mode. Wu et al. [27] explored the R&D competition and cooperation between a common supplier and two competing manufacturers, and established game models in which suppliers and manufacturers do not cooperate, suppliers cooperate with one manufacturer M1, suppliers cooperate with another manufacturer M2, and suppliers cooperate with both manufacturers. The influence of spillover rate, R&D efficiency and competition level on equilibrium solution is compared and analyzed. Sánchez-Sellero et al. [28] investigated the relationship between green innovation and research and development (R&D) is discussed. The results showed that internal and external R&D efforts promote green innovation. Zhu et al. [29] studied three green manufacturing modes in the government’s carbon tax policy, and found that the R&D difficulty of remanufacturing technology had an important influence on the choice of green manufacturing modes. Zhu et al. [30] studied the influence of government subsidies for green product R&D on competitive supply chain decision-making and found that R&D subsidies increased the profits of supply chain enterprises and consumer surplus. Khan et al. [31] applied a structural equation model to prove that green practices, supply chain crisis mitigation strategies, and intelligent technologies improved the performance of sustainable supply chains during the COVID-19 epidemic. Khan et al. [32] found that green capability plays a key role in green procurement, and green procurement behavior has a positive impact on economic and environmental performance. Liu et al. [33] investigated the specific role of supply chain capability in green operation, and whether the relationship between supply chain capability and green operation depends on environmental proactivity. The study found that there is a significant positive correlation between specific supply chain capability and green operation strategy, and environmental proactivity plays a regulatory role. Dai et al. [34] applied the theory of strategy-structure-capability-performance (SSCP) to build a model to describe how green cooperation with suppliers and green process innovation can make enterprises more competitive. Kumar et al. [35] confirmed that collaborative lean and green implementation can promote innovation practice through case studies and inductive theory construction methods. Lin et al. [36] discussed the relationship and influence between R&D subsidies (RDS) and green technology innovation (GTI) through empirical analysis and found that RDS and GTI of renewable energy enterprises showed an inverted U-shaped relationship, indicating that RDS has a promoting effect on GTI. Chen et al. [37] proposed a three-stage super-efficiency DEA model to study the R&D green innovation in China’s high-tech industry and evaluated the efficiency of R&D green innovation through empirical analysis. The results show that the efficiency of R&D green innovation is low and there is a lot of room for improvement. Lai et al. [38] studied the green innovation management practices of manufacturers applying digital transformation and explored the influencing factors of digital transformation of green supply chains on the performance improvement of manufacturers. Gu et al. [39] empirically tested the impact of the application of supply chain finance on green innovation by China’s A-share listed companies, and the study showed that the application of supply chain finance could significantly improve the output of green innovation and ease the pressure of capital. Wang et al. [40] analyzed green innovation in supply chain networks, and discussed the influence of supply chain network strength and cohesion on enterprises’ green innovation output and the regulatory role of enterprises’ environmental information disclosure. It is found that environmental information disclosure positively regulates the relationship between network power and green innovation output. Li et al. [41] studied the issue of green investment in sustainable supply chains and found that blockchain can improve the green sensitivity of customers, manufacturers have incentives to adopt blockchain, and fair retailers can encourage blockchain implementation. Peng et al. [42] studied the competition and cooperation strategies in green marketing of dual-channel supply chains considering customer satisfaction. The study found that under the influence of customer satisfaction, the optimal price is not always proportional to green marketing efforts, and retailers with higher market share do not necessarily enjoy higher profits. Assumpcao et al. [43] put forward the connection between eight different types of green supply chain management practices and enterprise innovation, and found that the internal heterogeneity of green supply chains management practices affects innovation. There was a strong relationship between innovation and the four categories of green supply chain management practices, but not with the other four. Shi et al. [44] considered four scenarios to determine who is more suitable to implement green product development or green marketing. It is found that there is a significant interaction between green product development and green marketing, which should be considered at the same time. Fontoura et al. [45] provided empirical evidence to analyze how supply chain leadership (SCL), supply chain follower (SCF), and Green Supply chain Integration (GSCI) contribute to green new product development (GNPD) and performance (PRF) through the impact of green innovation (GRI). To help better understand the impact of green innovation approaches on business sustainability. Lai et al. [46] studied two supply chain financing schemes of partial credit and bank trade credit, and found that manufacturers would reduce green investment even if suppliers provided financial support, and whether manufacturers chose internal or outsourcing financing depended on bank interest rates and the income sharing ratio between manufacturers and external financiers. Feng et al. [47] mentioned that green supply chain innovation (GSCI) is becoming a new paradigm of green supply chain management, which is gaining more and more attention in research and practice, and manufacturers adopted new technologies such as artificial intelligence and blockchain to improve green supply chain management. Xing et al. [48] studied how product service innovation can improve the environmental performance of green supply chains, and how to integrate product service innovation into green supply chain management from the perspective of product life cycle. Qu et al. [49] discussed the moderating effects of supplier integration and customer orientation on green innovation performance, and confirmed that supply chain integration has an important impact on promoting organizational green innovation. Dong et al. [50] analyzed the impact of green supply chain management on Chinese enterprises’ clean technology innovation and found that clean technology innovation benefits from green supply chain management. Feng et al. [51] discussed the selection strategy of green product R&D in the chain-to-chain competition environment, and applied two-pricing contracts to realize the coordination of supply chain. It was found that whether the supply chain carried out green product R&D would be affected by the competitive supply chain selection strategy and the cost of green product R&D. Zhang et al. [52] established a mixed integer programming model to study the optimal design of sustainable supply chain network, and put forward a chaotic particle-ant colony algorithm to solve multi-objective functions. Mohammadi et al. [53] established a multi-objective mixed integer nonlinear programming model for the sustainable supply chain of perishable products with price-dependent demand and deterioration rate, determined the optimal pricing strategy and cycle length, and achieved profit maximization and specific social goals. Fu et al. [54] discussed the influence of non-subsidy policy, fixed subsidy policy and agricultural risk coverage (ARC) subsidy policy on government costs, farmers and enterprise profits. It is found that the fixed subsidy policy and ARC policy improve the level of farmers’ environmentally sustainable investment and increase the profits of farmers and companies. Zhong et al. [55] constructed a fuzzy multi-objective optimization model to select suppliers of sustainable supply chains from three aspects: economy, environment and society.

As can be seen from the above literature, green innovation and green product R&D have been widely concerned by enterprises and scholars. It has been proved that green innovation and green product R&D have a significant positive impact on improving economic and environmental performance. Based on the above literatures, this paper studies the choice of green product R&D modes in different power structures to promote the green transformation of enterprises and provide theoretical support for green innovation and R&D of sustainable supply chains.

### 2.3 Coordination of sustainable supply chains

Each member in the supply chain system pursues the maximization of their interests, which will lead to the failure of the entire supply chain system to reach the optimal result. Therefore, it is necessary to coordinate the transfer payment among each member to make the profit of each member consistent with the optimal total profit target of the supply chain. In the field of contract coordination of supply chains, many scholars have carried out related research. Xu et al. [56] established centralized and decentralized decision-making models composed of a manufacturer and a retailer based on considering the impact of product environmental protection degree, and applied the revenue sharing contract to realize the coordination between manufacturers and retailers. Li et al. [57] studied the equilibrium results and coordination methods of the green supply chain, and constructed different game models of pricing strategy, profit coordination and information mode. Jiang et al. [58] investigated the manufacturer’s fairness preference, based on the product green level provided by suppliers as the reference standard, built a reward and penalty contract model based on the product green level, and analyzed the influence of manufacturer’s fairness preference on the product green level, price, manufacturer’s profit, supplier’s power and overall profit. Song et al. [59] studied the coordination of a three-level green fresh supply chain and constructed a joint contract of revenue and cost-sharing and a cross-joint contract model of cost and revenue sharing to improve the sustainable performance of the green supply chain.Wang et al. [60] established a supply chain model consisting of an e-commerce platform and a manufacturer, adopting Nash transaction contracts and Rubinstein negotiation contracts to mitigate benefit conflicts. The results showed that both the Nash contract and Rubinstein negotiation contract could realize the coordination of the supply chain and alleviate the conflicts of supply chain members. Chang et al. [61] studied the coordination between green supply chains constrained by capacity and green marketing efforts and designed a green marketing contract combining cost sharing and revenue sharing to coordinate the supply chain. The research showed that the cost-sharing and revenue-sharing contracts in green marketing can coordinate the supply chain. Chen et al. [62] explored the influence of CSR and consumer green preference on supply chain performance and product green level in dual-channel green supply chains. The results showed that cooperative contracts can coordinate the dual-channel supply chain and guarantee the profitability of enterprises. Ebrahimi et al. [63] proposed an incentive contract to coordinate environmental and social decisions of manufacturers and duopoly retailers in the green supply chain and proposed an environmental and social cost-sharing contract to encourage supply chain members to participate in the coordination model. The results showed that the coordination contract not only improved the profitability of the supply chain but also improves the green quality and CSR investment. Yang et al. [64] examined the coordination of green supply chains considering the reciprocity preference of retailers, and established and analyzed the dispersion models with and without reciprocity. On this basis, the cost-sharing joint commission contract is designed. The results showed that, within the reasonable range of retailer reciprocity preference, the higher the value of retailer reciprocity preference, the better the realization of environmental protection and the improvement of the economic well-being of the whole society. Cost-sharing contracts played a positive role in improving the environmental and economic performance of green supply chains. Mohsin et al. [65] used dynamic wholesale price contracts to coordinate the supply chain. and found that the dynamic wholesale price mechanism can realize supply chain coordination when the coordination parameters are within a certain range. Asghari et al. [66] studied the environmental responsibility of green closed-loop supply chain members and compared the economic and environmental performance of cost-sharing, benefit-sharing and two tariff coordination contracts, and found that the economic benefits in different contracts are different. Song et al. [67] discussed the pricing problem of selling both non-green products and substitutable green products and carried out supply chain coordination through a cost-sharing contract. The study found that cost-sharing contracts can coordinate the supply chain, and manufacturers and retailers can obtain higher profits. Qiao et al. [68] studied the coordination of sustainability supply chains considering the green consciousness of consumers. Studies have shown that green markets are more profitable and supply chain companies can achieve Pareto improvements in volume discount contracts. Jian et al. [69] designed the profit-sharing contract to coordinate the green closed-loop supply chain considering the fairness of manufacturers. The study found that profit-sharing contracts can improve the relationship between enterprises in the supply chain and achieve sustainable development of the economy and environment. Ghosh et al. [70] studied the coordination of sales price discount contracts on the trade credit strategy of the green supply chain and found that this contract can improve retailers’ sales efforts and product green level. Ranjan et al. [71] studied the pricing strategy and coordination mechanism of the dual-channel supply chain, realized channel coordination through the residual profit sharing mechanism, and achieved a win-win situation for supply chain enterprises. Liu et al. [72] proposed a revenue-sharing model to study the coordination of green supply chains in the dynamic adjustment pricing strategy and found that the coordination mechanism can improve the green level of products, corporate profits, consumer surplus, and social welfare. Tokta-palut [73] proposed a revenue-sharing contract based on Nash bargaining to coordinate the supply chain. The study found that, in terms of market demand and profitability, the classical supply chain with a higher degree of coordination can dominate the decentralized Industry 4.0 supply chain. Chen et al. [74] put forward a benefit-cost sharing contract to coordinate decentralized decision-making in sustainable supply chains considering both fair concern and risk aversion.

The above literatures use revenue-sharing contracts, cost-sharing contracts, wholesale price contracts, two-tariff contracts, collaboration contracts and cross-association contracts to realize the coordination of green supply chains. In this paper, two-part pricing contracts are used to coordinate the optimal modes of green product R&D in different power structures.

## 3. The problem description and model assume

This paper studies a three-level sustainable supply chain consisting of manufacturers, retailers, and third-party companies. Manufacturers sell their products to retailers, and retailers sell products to customers. Green product R&D can be conducted by manufacturers, retailers, enterprise alliances between manufacturers and retailers, and third-party companies, The different modes of green products R&D include manufacturers’ green product R&D, retailers’ green product R&D, manufacturers outsourcing green product R&D to third-party companies, retailers outsourcing green product R&D to third-party companies and manufacturers’ and retailers’ joint green products R&D. Relevant symbols involved in this paper and their meanings are shown in Table 1.

**Table 1:** Relevant symbols and their meanings.

Symbols	Meanings	Symbols	Meanings
**D**	Product demand function	**D**_**0**_	Basic demand for products
**α**	Sensitive coefficient of product prices	**γ**	Sensitive coefficient of green level
**p_1_**	The unit product price, p_1_ ≥ w_1_	**w**_**1**_	Wholesale price of the unit product, w_1_ ≥ c_1_
**c_1_**	The unit production cost, c_1_ ≥ 0	**u**_**1**_	Retailers’ unit net profits, u_1_ ≥ 0
**η**	Cost coefficient of green product R&D	**J**	Centralized decision-making
**F**	Fixed fees	**w**_**2**_	The outsourcing price of unit products
**π_r_**	Profits of retailers	**π**_**m**_	Profits of manufacturers
**π_t_**	Profits of third-party companies	**π**_**sc**_	Total profit of supply chains
**s_i_**	Green level of products in case i, i = m,r,t, m-manufacturers, r-retailers.t-third-party companies	**RR**	When retailers are core enterprises, retailers conduct green product R&D.
**MM**	When manufacturers are core enterprises, manufacturers conduct green product R&D.	**RM**	When retailers are core enterprises, manufacturers conduct green product R&D.
**MR**	When manufacturers are core enterprises, retailers conduct green product R&D.	**RC**	When retailers are core enterprises, retailers and manufacturers jointly conduct green product R&D.
**MC**	When manufacturers are core enterprises, retailers and manufacturers jointly conduct green product R&D.	**RMT**	When retailers are core enterprises, manufacturers outsource green product R&D to third-party companies.
**MMT**	When manufacturers are core enterprises, manufacturers outsource green product R&D to third-party companies.	**RRT**	When retailers are core enterprises, retailers outsource green product R&D to third-party companies.
**MRT**	When manufacturers are core enterprises, retailers outsource green product R&D to third-party companies.	**NMT**	When the power is equal, manufacturers outsource green product R&D to third-party companies.
**NR**	When the power is equal, retailers conduct green product R&D.	**NM**	When the power is equal, manufacturers conduct green product R&D.
**NC**	When the power is equal, retailers and manufacturers jointly conduct green product R&D.	**NRT**	When the power is equal, retailers outsource green product R&D to third-party companies.

The following hypotheses are made for the model:

**Hypothesis 1**. The demand function of green products is D = D_0_ − αp_1_ + γs_i_, i = m, r, t. When manufacturers conduct green product R&D, the demand function is D = D_0_ − αp_1_ + γs_m_. When retailers conduct green product R&D, the demand function is D = D_0_ − αp_1_ + γs_r_. When manufacturers and retailers jointly conduct green product R&D, the demand function is D = D_0_ − αp_1_ + γs_m_ + γs_r_. When manufacturers or retailers outsource green product R&D to third-party companies, the demand function is D = D_0_ − αp_1_ + γs_t_.
**Hypothesis 2**. The green R&D cost of enterprises is $\frac{1}{2}\eta s_{i}^{2},\;i=m,r,t$, among η represents the green R&D cost coefficient.

It can be obtained from the above hypotheses:

When the manufacturer conducts green product R&D, the profits of the manufacturer and the retailer are:

$$\pi_{m}=\left(w_{1}-c_{1}\right)(D_{0}-\alpha p_{1}+\gamma s_{m})-\frac{1}{2}\eta s_{m}^{2}$$ (1)

$$\pi_{r}=\left(p_{1}-w_{1}\right)\left(D_{0}-\alpha p_{1}+\gamma s_{m}\right)$$ (2)

When retailers conduct green product R&D, the profits of manufacturers and retailers are:

$$\pi_{m}=\left(w_{1}-c_{1}\right)(D_{0}-\alpha p_{1}+\gamma s_{r})$$ (3)

$$\pi_{r}=(p_{1}-w_{1})\;(D_{0}-\alpha p_{1}+\gamma s_{r})-\frac{1}{2}\eta s_{r}^{2}$$ (4)

When manufacturers outsource green product R&D to third-party companies, the profits of manufacturers, retailers, and third-party companies are:

$$\pi_{m}=\left(w_{1}-w_{2}-c_{1}\right)\left(D_{0}-\alpha p_{1}+\gamma s_{t}\right)$$ (5)

$$\pi_{r}=\left(p_{1}-w_{1}\right)\left(D_{0}-\alpha p_{1}+\gamma s_{t}\right)$$ (6)

$$\pi_{t}=w_{2}\left(D_{0}-\alpha p_{1}+\gamma s_{t}\right)-\frac{1}{2}\eta s_{t}^{2}$$ (7)

When retailers outsource green product R&D to third-party companies, the profits of manufacturers, retailers and third-party companies are:

$$\pi_{m}=\left(w_{1}-c_{1}\right)\left(D_{0}-\alpha p_{1}+\gamma s_{t}\right)$$ (8)

$$\pi_{r}=\left(p_{1}-w_{1}-w_{2}\right)\left(D_{0}-\alpha p_{1}+\gamma s_{t}\right)$$ (9)

$$\pi_{t}=w_{2}\left(D_{0}-\alpha p_{1}+\gamma s_{t}\right)-\frac{1}{2}\eta s_{t}^{2}$$ (10)

When manufacturers and retailers jointly conduct green product R&D, the profits of manufacturers and retailers are:

$$\pi_{m}=\left(w_{1}-c_{1}\right)\left(D_{0}-\alpha p_{1}+\gamma s_{m}+\gamma s_{r}\right)-\frac{1}{2}\eta s_{m}^{2}$$ (11)

$$\pi_{r}=\left(p_{1}-w_{1}\right)\left(D_{0}-\alpha p_{1}+\gamma s_{m}+\gamma s_{r}\right)-\frac{1}{2}\eta s_{r}^{2}$$ (12)

## 4. Game models of green product R&D modes

### 4.1 Manufacturers are core enterprises

#### 4.1.1Manufacturers conduct green product R&D (MM)

When manufacturers conduct green product R&D, because manufacturers are core enterprises, manufacturers first determine the wholesale price and green level of products, and then retailers determine the retail price of products. According to the reverse induction method, let p_1_ = w_1_ + U_1_, we obtain $p_{1}^{MM}=\frac{D_{0}+\alpha w_{1}+\gamma s_{m}}{2\alpha }$ from $\frac{\partial \pi_{r}}{\partial p_{1}}=0$. Substitute $p_{1}^{MM}$ into π_m_, and get $w_{1}^{MM}=\frac{D_{0}+\alpha c_{1}+\gamma s_{m}}{2\alpha }$, $s_{m}^{MM}=\frac{\left(w_{1}-c_{1}\right)\gamma }{2\eta }$ from $\frac{\partial \pi_{m}}{\partial w_{1}}=0,\;\frac{\partial \pi_{m}}{\partial s_{m}}=0$. Simultaneous solution:$w_{1}^{MM}=\frac{{2D}_{0}\eta +\left(2\alpha \eta -\gamma^{2}\right)c_{1}}{4\alpha \eta -\gamma^{2}}$, $s_{m}^{MM}=\frac{{(D}_{0}-\alpha c_{1})\gamma }{4\alpha \eta -\gamma^{2}}$, $p_{1}^{MM}=\frac{3D_{0}\eta +\left(\alpha \eta -\gamma^{2}\right)c_{1}}{4\alpha \eta -\gamma^{2}}$. So we can obtain $D^{MM}=\frac{{(D}_{0}-\alpha c_{1})\alpha \eta }{4\alpha \eta -\gamma^{2}}$, ${\;\pi }_{m}^{MM}=\frac{{{(D}_{0}-\alpha c_{1})}^{2}\eta }{2\left(4\alpha \eta -\gamma^{2}\right)}$, $\pi_{r}^{MM}=\frac{{{(D}_{0}-\alpha c_{1})}^{2}\alpha \eta^{2}}{{\left(4\alpha \eta -\gamma^{2}\right)}^{2}}$, $\pi_{sc}^{MM}=\frac{{{(6\alpha \eta -\gamma^{2})(D}_{0}-\alpha c_{1})}^{2}\eta }{{2\left(4\alpha \eta -\gamma^{2}\right)}^{2}}$.

#### 4.1.2 Retailers conduct green product R&D (MR)

When retailers conduct green product R&D, because manufacturers are the core enterprise, manufacturers first determine the wholesale price of the product, and retailers then determine the retail price and the green level of products. According to the reverse induction method, we can get $p_{1}^{MR}=\frac{D_{0}+\alpha w_{1}+\gamma s_{r}}{2\alpha }$, ${\;s}_{r}^{MR}=\frac{(p_{1}-w_{1})\gamma }{\eta }$ from $\frac{\partial \pi_{r}}{\partial p_{1}}=0,\;\frac{\partial \pi_{r}}{\partial s_{r}}=0$. Through simultaneous solution and obtain: ${\text{p}}_{\text{1}}^{\text{MR}}=\frac{D_{0}\eta +(\alpha \eta -\gamma^{2}{)w}_{1}}{\text{2}\alpha \eta -\gamma^{2}}$, $s_{r}^{MR}=\frac{\left(D_{0}-\alpha w_{1}\right)\gamma }{\text{2}\alpha \eta -\gamma^{2}}$. Substitute $p_{1}^{MR}$, ${\;s}_{r}^{MR}$ into π_m_, and obtain $w_{1}^{MR}=\frac{D_{0}+\alpha c_{1}}{2\alpha }$ from $\frac{\partial \pi_{m}}{\partial w_{1}}=0$, then ${\text{p}}_{\text{1}}^{\text{MR}}=\frac{\text{(3}\alpha \eta -\gamma^{2}\text{)}D_{0}\text{+(}\alpha \eta -\gamma^{2}\text{)}\alpha c_{1}}{2\alpha (2\alpha \eta -\gamma^{2})}$, $s_{r}^{MR}=\frac{{(D}_{0}-\alpha c_{1}\text{)}\gamma }{\text{2(2}\alpha \eta -\gamma^{2})}$, we can obtain ${\text{D}}^{MR}=\frac{{(D}_{0}-\alpha c_{1})\alpha \eta }{\text{2(2}\alpha \eta -\gamma^{2})}$, $\pi_{m}^{MR}=\frac{{\left(D_{0}-\alpha c_{1}\right)}^{2}\eta }{4(2\alpha \eta -\gamma^{2})}$, $\pi_{r}^{MR}=\frac{{{(D}_{0}-\alpha c_{1})}^{2}\eta }{8\left(2\alpha \eta -\gamma^{2}\right)}$, $\pi_{sc}^{MR}=\frac{{{3(D}_{0}-\alpha c_{1})}^{2}\eta }{8\left(2\alpha \eta -\gamma^{2}\right)}$.

#### 4.1.3 Manufacturers outsource green product R&D to third-party enterprises (MMT)

When manufacturers outsource green product R&D to third-party enterprises, manufacturers first determine the wholesale price w_1_ because manufacturers are core enterprises, then retailers and third-party companies make decisions at the same time. Retailers decide the retail price of products, and third-party companies decide the wholesale price w_2_ and the green level of products.

Assuming p_1_ = w_1_ + w_2_ + U_1_, according to the inverse induction method, from $\frac{\partial \pi_{t}}{\partial w_{2}}=0$, $\frac{\partial \pi_{t}}{\partial s_{t}}=0$, $w_{2}^{MMT}=\frac{D_{0}-\alpha p_{1}+\gamma s_{t}}{\alpha }$, $s_{t}^{MMT}=\frac{w_{2}\gamma }{\eta }$. From $\frac{\partial \pi_{r}}{\partial p_{1}}=0$, $p_{1}^{MMT}=\frac{D_{0}+\gamma s_{t}+\alpha w_{1}}{2\alpha }$. By simultaneous solution, we can get $w_{2}^{MMT}=\frac{{(D}_{0}-\alpha w_{1})\eta }{2\alpha \eta -\gamma^{2}}$, $s_{t}^{MMT}=\frac{{(D}_{0}-\alpha w_{1})\gamma }{2\alpha \eta -\gamma^{2}}$. Substitute $p_{1}^{MMT}$, $w_{2}^{MMT}$ and ${\;s}_{t}^{MMT}$ into π_m_, from $\frac{\partial \pi_{m}}{\partial w_{1}}=0$, $w_{1}^{MMT}=\frac{\left(4\alpha \eta -\gamma^{2}\right)D_{0}+\left(2\alpha \eta -\gamma^{2}\right)\alpha c_{1}}{2\alpha (3\alpha \eta -\gamma^{2})}$. We can obtain $w_{2}^{MMT}=\frac{{(D}_{0}-\alpha c_{1})\eta }{2(3\alpha \eta -\gamma^{2})}$, $s_{t}^{MMT}=\frac{{(D}_{0}-\alpha c_{1})\gamma }{2(3\alpha \eta -\gamma^{2})}$, $p_{1}^{MMT}=\frac{\left(5\alpha \eta -\gamma^{2}\right)D_{0}+\left(\alpha \eta -\gamma^{2}\right)\alpha c_{1}}{2\alpha (3\alpha \eta -\gamma^{2})}$, $D^{MMT}=\frac{{(D}_{0}-\alpha c_{1})\alpha \eta }{2(3\alpha \eta -\gamma^{2})}$, $\pi_{m}^{MMT}=\frac{{{(D}_{0}-\alpha c_{1})}^{2}\eta }{4(3\alpha \eta -\gamma^{2})}$, $\pi_{r}^{MMT}=\frac{{{(D}_{0}-\alpha c_{1})}^{2}\alpha \eta^{2}}{4{\left(3\alpha \eta -\gamma^{2}\right)}^{2}}$, $\pi_{t}^{MMT}=\frac{{{\left(2\alpha \eta -\gamma^{2}\right)(D}_{0}-\alpha c_{1})}^{2}\eta }{8{\left(3\alpha \eta -\gamma^{2}\right)}^{2}}$, $\pi_{sc}^{MMT}=\frac{{{\left(10\alpha \eta -3\gamma^{2}\right)(D}_{0}-\alpha c_{1})}^{2}\eta }{8{\left(3\alpha \eta -\gamma^{2}\right)}^{2}}$.

#### 4.1.4 Retailers outsource green product R&D to third-party enterprises (MRT)

When retailers outsource green product R&D to third-party enterprises, manufacturers first determine the wholesale price w_1_ because manufacturers are core enterprises, then retailers and third-party companies make decisions at the same time. Retailers decide the retail price of products, and third-party companies decide the wholesale price w_2_ and the green level of products.

Assuming p_1_ = w_1_ + w_2_ + U_1_, according to the inverse induction method, from $\frac{\partial \pi_{t}}{\partial w_{2}}=0$, $\frac{\partial \pi_{t}}{\partial s_{t}}=0$, $w_{2}^{MRT}=\frac{D_{0}-\alpha p_{1}+\gamma s_{t}}{\alpha }$, $s_{t}^{MRT}=\frac{w_{2}\gamma }{\eta }$. From $\frac{\partial \pi_{r}}{\partial p_{1}}=0$, $p_{1}^{MRT}=\frac{D_{0}+\gamma s_{t}+\alpha w_{1}+\alpha w_{2}}{2\alpha }$. By simultaneous solution, we can get $w_{2}^{MRT}=\frac{{(D}_{0}-\alpha w_{1})\eta }{3\alpha \eta -\gamma^{2}}$, $s_{t}^{MRT}=\frac{{(D}_{0}-\alpha w_{1})\gamma }{3\alpha \eta -\gamma^{2}}$. Substitute $p_{1}^{MRT}$, $w_{1}^{MRT}$ and $s_{t}^{MRT}\;$ into π_m_, from $\frac{\partial \pi_{m}}{\partial w_{1}}=0$, $w_{1}^{MRT}=\frac{D_{0}+\alpha c_{1}}{2\alpha }$. We can obtain $w_{2}^{MRT}=\frac{{(D}_{0}-\alpha c_{1})\eta }{2(3\alpha \eta -\gamma^{2})}$, $s_{t}^{MRT}=\frac{{(D}_{0}-\alpha c_{1})\gamma }{2(3\alpha \eta -\gamma^{2})}$, $p_{1}^{MRT}=\frac{\left(5\alpha \eta -\gamma^{2}\right)D_{0}+\left(\alpha \eta -\gamma^{2}\right)\alpha c_{1}}{2\alpha (3\alpha \eta -\gamma^{2})}$, $D^{MRT}=\frac{{(D}_{0}-\alpha c_{1})\alpha \eta }{2(3\alpha \eta -\gamma^{2})}$, $\pi_{m}^{MRT}=\frac{{{(D}_{0}-\alpha c_{1})}^{2}\eta }{4(3\alpha \eta -\gamma^{2})}$, ${\;\pi }_{r}^{MRT}=\frac{{{(D}_{0}-\alpha c_{1})}^{2}\alpha \eta^{2}}{4{\left(3\alpha \eta -\gamma^{2}\right)}^{2}}$, $\pi_{t}^{MRT}=\frac{{{\left(2\alpha \eta -\gamma^{2}\right)(D}_{0}-\alpha c_{1})}^{2}\eta }{8{\left(3\alpha \eta -\gamma^{2}\right)}^{2}}$, $\pi_{sc}^{MRT}=\frac{{{\left(10\alpha \eta -3\gamma^{2}\right)(D}_{0}-\alpha c_{1})}^{2}\eta }{8{\left(3\alpha \eta -\gamma^{2}\right)}^{2}}$.

#### 4.1.5 Manufacturers and retailers jointly conduct green product R&D (MC)

When manufacturers and retailers jointly conduct green product R&D, since manufacturers are core enterprises, manufacturers first determine the wholesale price of products and the green level of products, then retailers determine the retail price of products and the green level of products. According to the reverse induction method, it can be obtained $p_{1}^{MC}=\frac{D_{0}+\alpha w_{1}+\gamma s_{m}+\gamma s_{r}}{2\alpha }$, $s_{r}^{MC}=\frac{(p_{1}-w_{1})\gamma }{\eta }$ from $\frac{\partial \pi_{r}}{\partial p_{1}}=0$, $\frac{\partial \pi_{r}}{\partial s_{r}}=0$. Simultaneous solution can be obtained: $w_{1}^{MC}=\frac{D_{0}(2\alpha \eta -\gamma^{2})+\alpha c_{1}(2\alpha \eta -2\gamma^{2})}{\alpha (4\alpha \eta -3\gamma^{2})}$, $s_{m}^{MC}=\frac{\left(D_{0}-\alpha c_{1}\right)\gamma }{4\alpha \eta -3\gamma^{2}}$, ${\text{p}}_{\text{1}}^{\text{MC}}=\frac{D_{0}(\text{3}\alpha \eta -\gamma^{2}\text{)}+(\alpha \eta -2\gamma^{2})\alpha c_{1}}{\alpha (4\alpha \eta -3\gamma^{2})}$, $s_{r}^{MC}=\frac{\left(D_{0}-\alpha c_{1}\right)\gamma }{\text{4}\alpha \eta -3\gamma^{2}}$. We can obtain $D^{MC}=\frac{{(D}_{0}-\alpha c_{1})\alpha \eta }{4\alpha \eta -3\gamma^{2}}$, $\pi_{m}^{MC}=\frac{{\left(D_{0}-\alpha c_{1}\right)}^{2}\eta }{\text{2(4}\alpha \eta -3\gamma^{2})}$, $\pi_{r}^{MC}=\frac{{\text{(}D_{0}-\alpha c_{1})}^{2}(2\alpha \eta -\gamma^{2})\eta }{\text{2}{\text{(4}\alpha \eta -3\gamma^{2})}^{2}}$, $\pi_{sc}^{MC}=\frac{{\text{(}D_{0}-\alpha c_{1})}^{2}(3\alpha \eta -2\gamma^{2})\eta }{{\text{(4}\alpha \eta -3\gamma^{2})}^{2}}$.

**Theorem 1**. When 2αη − γ^2^ > 0, $D^{MR}\geq D^{MM},\;s_{r}^{MR}\geq s_{m}^{MM},\pi_{m}^{MR}\geq \pi_{m}^{MM},\pi_{r}^{MR}{\geq \pi }_{r}^{MM}$. When αη − γ^2^ ≥ 0, ${\text{p}}_{\text{1}}^{\text{MR}}\leq {\text{p}}_{\text{1}}^{\text{MM}}$.

The proof of Theorem 1 is enclosed in the Appendix.

Theorem 1 states that when manufacturers are core enterprises, the market demand, product green level, manufacturers’ profits, and retailers’ profits for retailers to conduct green product R&D are greater than the optimal value for manufacturers to conduct green product R&D, and the retail price of green products is lower than the retail price for manufacturers to conduct green R&D.

**Theorem 2**. When 4αη − 3γ^2^ > 0, $D^{MC}\geq D^{MR},\;s_{r}^{MC}\geq s_{r}^{MR},\pi_{m}^{MC}\geq \pi_{m}^{MR},\pi_{r}^{MC}{\geq \pi }_{r}^{MR},{\text{p}}_{\text{1}}^{\text{MC}}\leq {\text{p}}_{\text{1}}^{\text{MR}}$.

The proof of Theorem 2 is enclosed in the Appendix.

Theorem 2 illustrates that when manufacturers are core enterprises, the market demand, product green level, manufacturers’ profits and retailers ’ profits for manufacturers and retailers to conduct jointly green product R&D are greater than the optimal value for retailers to conduct green product R&D, and the retail price of green products is lower than the retail price for retailers to conduct green product R&D.

**Theorem 3**. When 2αη − γ^2^ ≥ 0, $D^{MM}\geq D^{MMT},\;s_{m}^{MM}\geq s_{t}^{MMT},\;\pi_{m}^{MM}\geq \pi_{m}^{MMT},\pi_{r}^{MM}\geq \pi_{r}^{MMT}$. When αη − γ^2^ ≥ 0, ${\text{p}}_{\text{1}}^{\text{MM}}\leq {\text{p}}_{\text{1}}^{\text{MMT}}$.

The proof of Theorem 3 is enclosed in the Appendix.

Theorem 3 states that when manufacturers are core enterprises, the market demand, product green level, manufacturers’ profits and retailers’ profits for manufacturers to conduct green product R&D are greater than the optimal value for manufacturers to outsource green product R&D to third-party companies, and the retail price of green products is lower than the retail price for manufacturers to outsource green product R&D to third-party companies.

**Theorem 4**. When 2αη − γ^2^ > 0, $D^{MR}\geq D^{MRT},\;s_{m}^{MR}\geq s_{t}^{MRT},\;\pi_{m}^{MR}\geq \pi_{m}^{MRT},\pi_{r}^{MR}\geq \pi_{r}^{MRT}$. When αη − γ^2^ ≥ 0, ${\text{p}}_{\text{1}}^{\text{MR}}\leq {\text{p}}_{\text{1}}^{\text{MRT}}$.

The proof of Theorem 4 is enclosed in the Appendix.

Theorem 4 shows that when manufacturers are core enterprises, the market demand, product green level, manufacturers’ profits and retailers’ profits for retailers to conduct green product R&D are greater than the optimal value for retailers to outsource green product R&D to third-party companies, and the retail price of green products is lower than the retail price for retailers to outsource green product R&D to third-party companies.

From Theorems 1–4, we can see that when manufacturers are core enterprises, retailers’ green product R&D is better than that of manufacturers. The joint green product R&D of manufacturers and retailers is better than that of manufacturers or retailers. Manufacturers’ green product R&D is better than manufacturers outsourcing green product R&D to third-party companies, and retailers’ green product R&D is better than retailers outsourcing green product R&D to third-party companies.

### 4.2 Retailers are core enterprises

#### 4.2.1 Manufacturers conduct green product R&D (RM)

When retailers are core enterprises, retailers first determine the retail price of products, then manufacturers determine the wholesale price and green level of products. Let p_1_ = w_1_ + U_1_, according to the reverse induction method, from $\frac{\partial \pi_{m}}{\partial w_{1}}=0$, $\frac{\partial \pi_{m}}{\partial s_{m}}=0$, $w_{1}^{RM}=\frac{D_{0}-\alpha p_{1}+\gamma s_{m}+\alpha c_{1}}{\alpha }$, ${\;s}_{m}^{RM}=\frac{(w_{1}-c_{1})\gamma }{\eta },\;$ simultaneous solution can be obtained:$w_{1}^{RM}=\frac{{(D}_{0}-\alpha p_{1})\eta }{\alpha \eta -\gamma^{2}}+c_{1}$, $s_{m}^{RM}=\frac{(D_{0}-\alpha p_{1})\gamma }{\alpha \eta -\gamma^{2}}$. Substitute $w_{1}^{RM}$, ${\;s}_{r}^{MR}$ into π_r_, and obtain ${\text{p}}_{\text{1}}^{\text{RM}}=\frac{\text{(3}\alpha \eta -\gamma^{2}\text{)}D_{0}\text{+(}\alpha \eta -\gamma^{2}\text{)}\alpha c_{1\;}}{2\alpha (2\alpha \eta -\gamma^{2})}$ from $\frac{\partial \pi_{r}}{\partial p_{1}}=0$, then ${\text{w}}_{\text{1}}^{\text{RM}}=\frac{D_{0}\eta \text{+(3}\alpha \eta -2\gamma^{2}\text{)}c_{1}}{\text{2(2}\alpha \eta -\gamma^{2})}$, $s_{m}^{RM}=\frac{(D_{0}-\alpha c_{1})\gamma }{2(2\alpha \eta -\gamma^{2})}$. Substitute ${\text{p}}_{\text{1}}^{\text{RM}}$, ${\text{w}}_{\text{1}}^{\text{RM}}$, $s_{r}^{MR}$, we can obtain ${\text{D}}^{\text{RM}}=\frac{(D_{0}-\alpha c_{1})\alpha \eta }{\text{2(2}\alpha \eta -\gamma^{2})}$, $\pi_{m}^{RM}=\frac{{{(D}_{0}-\alpha c_{1})}^{2}\eta }{8\left(2\alpha \eta -\gamma^{2}\right)}$, $\pi_{r}^{RM}=\frac{{\left(D_{0}-\alpha c_{1}\right)}^{2}\eta }{\text{4(2}\alpha \eta -\gamma^{2})}$, $\pi_{sc}^{RM}=\frac{{{3(D}_{0}-\alpha c_{1})}^{2}\eta }{8\left(2\alpha \eta -\gamma^{2}\right)}$.

#### 4.2.2 Retailers conduct green product R&D (RR)

Similarly, retailers first determine the retail price and green level of products, then manufacturers decide the wholesale price of products. According to the reverse induction method, let p_1_ = w_1_ + U_1_, and get $w_{1}^{RR}=\frac{D_{0}+\alpha c_{1}-\alpha p_{1}+\gamma s_{r}}{\alpha }$ from $\frac{\partial \pi_{m}}{\partial w_{1}}=0$. Substitute $w_{1}^{RR}$ into π_r_, according to $\frac{\partial \pi_{r}}{\partial p_{1}}=0$, $\frac{\partial \pi_{r}}{\partial s_{r}}=0$, then $p_{1}^{RR}=\frac{\text{3}D_{0}\text{+3}\gamma s_{r}+\alpha c_{1}}{4\alpha }$, $s_{r}^{RR}=\frac{\left(3\alpha p_{1}-\text{2}D_{0}-\alpha c_{1}\right)\gamma }{2\gamma^{2}+\alpha \eta }$, simultaneous solution can be obtained: $p_{1}^{RR}=\frac{{3D}_{0}\eta +(\alpha \eta -\gamma^{2})c_{1}}{4\alpha \eta -\gamma^{2}}$, $s_{r}^{RR}=\frac{(D_{0}-\alpha c_{1})\gamma }{4\alpha \eta -\gamma^{2}}$, $w_{1}^{RR}=\frac{D_{0}\eta +(3\alpha \eta -\gamma^{2})c_{1}}{4\alpha \eta -\gamma^{2}}$. We can get $D^{RR}=\frac{{(D}_{0}-\alpha c_{1})\alpha \eta }{4\alpha \eta -\gamma^{2}}$, $\pi_{m}^{RR}=\frac{{{(D}_{0}-\alpha c_{1})}^{2}\alpha \eta^{2}}{{\left(4\alpha \eta -\gamma^{2}\right)}^{2}}$, $\pi_{r}^{RR}=\frac{{{(D}_{0}-\alpha c_{1})}^{2}\eta }{2\left(4\alpha \eta -\gamma^{2}\right)}$, $\pi_{sc}^{RR}=\frac{{{(D}_{0}-\alpha c_{1})}^{2}\left(6\alpha \eta -\gamma^{2}\right)\alpha \eta^{2}}{{2(4\alpha \eta -\gamma^{2})}^{2}}$.

#### 4.2.3 Manufacturers outsource green product R&D to third-party enterprises (RMT)

When manufacturers outsource green product R&D to third-party enterprises, retailers first determine the retail price because retailers are core enterprises, then manufacturers and third-party companies make decisions at the same time. Manufacturers decide the wholesale price w_1_ of products, and third-party companies decide the wholesale price w_2_ and the green level of products.

Assuming p_1_ = w_1_ + w_2_ + U_1_, according to the inverse induction method, from $\frac{\partial \pi_{t}}{\;\partial w_{2}}=0$, $\frac{\partial \pi_{t}}{\partial s_{t}}=0$, $w_{2}^{RMT}=\frac{D_{0}-\alpha p_{1}+\gamma s_{t}}{\alpha }$, $s_{t}^{MMT}=\frac{w_{2}\gamma }{\eta }$. From $\frac{\partial \pi_{m}}{\partial w_{1}}=0$, $w_{1}^{RMT}=\frac{{\text{D}}_{\text{0}}-\alpha p_{1}+\gamma s_{t}+\alpha w_{2}+\alpha c_{1}}{\alpha }$. Substitute $w_{1}^{RMT}$ and $s_{t}^{RMT}\;$ into π_r_, from $\frac{\partial \pi_{r}}{\partial p_{1}}=0$, $p_{1}^{RMT}=\frac{\left(5\alpha \eta -\gamma^{2}\right)D_{0}+\left(\alpha \eta -\gamma^{2}\right)\alpha c_{1}}{2\alpha (3\alpha \eta -\gamma^{2})}$. Then $w_{2}^{RMT}=\frac{{(D}_{0}-\alpha c_{1})\eta }{2(3\alpha \eta -\gamma^{2})}$, $s_{t}^{RMT}=\frac{{(D}_{0}-\alpha c_{1})\gamma }{2(3\alpha \eta -\gamma^{2})}$, $w_{1}^{RMT}=\frac{\left(D_{0}+2\alpha c_{1}\right)\eta -\gamma^{2}c_{1}}{3\alpha \eta -\gamma^{2}}$, $D^{RMT}=\frac{{(D}_{0}-\alpha c_{1})\alpha \eta }{2(3\alpha \eta -\gamma^{2})}$, $\pi_{m}^{RMT}=\frac{{{(D}_{0}-\alpha c_{1})}^{2}\alpha \eta^{2}}{4{\left(3\alpha \eta -\gamma^{2}\right)}^{2}}$, ${\;\pi }_{r}^{RMT}=\frac{{{(D}_{0}-\alpha c_{1})}^{2}\eta }{4(3\alpha \eta -\gamma^{2})}$, $\pi_{t}^{RMT}=\frac{{{\left(2\alpha \eta -\gamma^{2}\right)(D}_{0}-\alpha c_{1})}^{2}\eta }{8{\left(3\alpha \eta -\gamma^{2}\right)}^{2}}$, $\pi_{sc}^{RMT}=\frac{{{\left(10\alpha \eta -3\gamma^{2}\right)(D}_{0}-\alpha c_{1})}^{2}\eta }{8{\left(3\alpha \eta -\gamma^{2}\right)}^{2}}$.

#### 4.2.4 Retailers outsource green product R&D to third-party enterprises (RRT)

When retailers outsource green product R&D to third-party enterprises, retailers first determine the retail price because retailers are core enterprises, and then manufacturers and third-party companies make decisions at the same time. Manufacturers decide the wholesale price w_1_ of products, and third-party companies decide the wholesale price w_2_ and the green level of products.

Assuming p_1_ = w_1_ + w_2_ + U_1_, according to the inverse induction method, from $\frac{\partial \pi_{t}}{\;\partial w_{2}}=0$, $\frac{\partial \pi_{t}}{\partial s_{t}}=0$, ${\;w}_{2}^{RRT}=\frac{D_{0}-\alpha p_{1}+\gamma s_{t}}{\alpha }$, $s_{t}^{RRT}=\frac{w_{2}\gamma }{\eta }$. From $\frac{\partial \pi_{m}}{\partial w_{1}}=0$, $w_{1}^{RRT}=\frac{D_{0}-\alpha p_{1}+\gamma s_{t}+\alpha c_{1}}{\alpha }$. Substitute $w_{1}^{RRT}$, ${\;w}_{2}^{RRT}$ and $s_{t}^{RRT}$ into π_r_, from $\frac{\partial \pi_{r}}{\partial p_{1}}=0$, $p_{1}^{RRT}=\frac{\left(5\alpha \eta -\gamma^{2}\right)D_{0}+\left(\alpha \eta -\gamma^{2}\right)\alpha c_{1}}{2\alpha (3\alpha \eta -\gamma^{2})}$. Then $w_{2}^{RRT}=\frac{{(D}_{0}-\alpha c_{1})\eta }{2(3\alpha \eta -\gamma^{2})}$, $s_{t}^{RRT}=\frac{{(D}_{0}-\alpha c_{1})\gamma }{2(3\alpha \eta -\gamma^{2})}$, $w_{1}^{RRT}=\frac{\left(D_{0}+5\alpha c_{1}\right)\eta -{2\gamma }^{2}c_{1}}{2(3\alpha \eta -\gamma^{2})}$, $D^{RRT}=\frac{{(D}_{0}-\alpha c_{1})\alpha \eta }{2(3\alpha \eta -\gamma^{2})}$, $\pi_{m}^{RRT}=\frac{{{(D}_{0}-\alpha c_{1})}^{2}\alpha \eta^{2}}{4{\left(3\alpha \eta -\gamma^{2}\right)}^{2}}$, $\pi_{r}^{RRT}=\frac{{{(D}_{0}-\alpha c_{1})}^{2}\eta }{4(3\alpha \eta -\gamma^{2})}$, $\pi_{t}^{RMT}=\frac{{{\left(2\alpha \eta -\gamma^{2}\right)(D}_{0}-\alpha c_{1})}^{2}\eta }{8{\left(3\alpha \eta -\gamma^{2}\right)}^{2}}$, $\pi_{sc}^{RMT}=\frac{{{\left(10\alpha \eta -3\gamma^{2}\right)(D}_{0}-\alpha c_{1})}^{2}\eta }{8{\left(3\alpha \eta -\gamma^{2}\right)}^{2}}$.

#### 4.2.5 Manufacturers and retailers jointly conduct green product R&D (RC)

When manufacturers and retailers jointly conduct green product R&D, because retailers are core enterprises, retailers first determine the retail price and the green level of products, then manufacturers determine the wholesale price and green level of products. According to the reverse induction method, let p_1_ = w_1_ + u_1_, and get $w_{1}^{RC}=\frac{D_{0}-\alpha p_{1}+\gamma s_{m}+\gamma s_{r}+\alpha c_{1}}{\alpha }$, $s_{m}^{RC}=\frac{\left(w_{1}-c_{1}\right)\gamma }{\eta }\;$ by $\frac{\partial \pi_{m}}{\partial w_{1}}=0$, $\frac{\partial \pi_{m}}{\partial s_{m}}=0$, simultaneous solution can be obtained: $w_{1}^{RC}=\frac{{\text{D}}_{\text{0}}-\alpha p_{1}+\gamma s_{r}+\alpha c_{1}(\alpha \eta -\gamma^{2})}{\alpha (\alpha \eta -\gamma^{2})}$, $s_{m}^{RC}=\frac{\left(D_{0}-\alpha p_{1}+\gamma s_{r}\right)\gamma }{\alpha \eta -\gamma^{2}}$. Substitute $w_{1}^{RC}$, $s_{m}^{RC}$ into π_r_, from $\frac{\partial \pi_{r}}{\partial p_{1}}=0$, $\frac{\partial \pi_{r}}{\partial s_{r}}=0$, we can obtain $p_{1}^{RC}=\frac{D_{0}\left(3\alpha \eta -\gamma^{2}\right)+\gamma s_{r}\left(3\alpha \eta -\gamma^{2}\right)+\alpha c_{1}(\alpha \eta -\gamma^{2})}{2\alpha (2\alpha \eta -\gamma^{2})}$, $s_{r}^{RC}=\frac{[\alpha p_{1}\left(3\alpha \eta -\gamma^{2}\right)-2D_{0}\alpha \eta -\alpha c_{1}(\alpha \eta -\gamma^{2})]\gamma }{\alpha^{2}\eta^{2}+\gamma^{4}}$. Simultaneous solution can be obtained: ${\text{p}}_{\text{1}}^{\text{RC}}=\frac{D_{0}(\text{3}\alpha \eta -\gamma^{2}\text{)}+\alpha c_{1}(\alpha \eta -2\gamma^{2})}{\alpha (4\alpha \eta -3\gamma^{2})}$, $s_{r}^{RC}=\frac{\left(D_{0}-\alpha c_{1}\right)\gamma }{4\alpha \eta -3\gamma^{2}}$, $w_{1}^{RC}=\frac{D_{0}\eta +(3\alpha \eta -3\gamma^{2})c_{1}}{4\alpha \eta -3\gamma^{2}}$, $s_{m}^{RC}=\frac{\left(D_{0}-\alpha c_{1}\right)\gamma }{4\alpha \eta -3\gamma^{2}}$. Then $D^{RC}=\frac{{(D}_{0}-\alpha c_{1})\alpha \eta }{4\alpha \eta -3\gamma^{2}}$, $\pi_{m}^{RC}=\frac{{\text{(}D_{0}-\alpha c_{1})}^{2}(2\alpha \eta -\gamma^{2})\eta }{\text{2}{\text{(4}\alpha \eta -3\gamma^{2})}^{2}}$, ${\;\pi }_{r}^{RC}=\frac{{\text{(}D_{0}-\alpha c_{1})}^{2}\eta }{\text{2(4}\alpha \eta -3\gamma^{2})}$, ${\;\pi }_{sc}^{RC}=\frac{{\text{(}D_{0}-\alpha c_{1})}^{2}(3\alpha \eta -2\gamma^{2})\alpha \eta^{2}}{{\text{(4}\alpha \eta -3\gamma^{2})}^{2}}$.

**Theorem 5**. When 2αη − γ^2^ > 0, $D^{RM}\geq D^{RR},\;s_{r}^{RM}\geq s_{m}^{RR},\;\pi_{m}^{RM}\geq \pi_{m}^{RR},\;\pi_{r}^{RM}{\geq \pi }_{r}^{RR}$. When αη − γ^2^ ≥ 0, ${\text{p}}_{\text{1}}^{\text{RR}}\leq {\text{p}}_{\text{1}}^{\text{RM}}$.

The proof of Theorem 5 is enclosed in the Appendix.

Theorem 5 states that when retailers are core enterprises, the market demand, product green level, manufacturers’ profits, and retailers’ profits for manufacturers to conduct green product R&D are greater than the optimal value for retailers to conduct green product R&D, and the retail price of green products is lower than the retail price for retailers to conduct green R&D.

**Theorem 6**. When 4αη − 3γ^2^ > 0, D^RC^ ≥ D^RM^, $s_{r}^{RC}\geq s_{r}^{RM},\;\pi_{m}^{RC}\geq \pi_{m}^{RM},\;\pi_{r}^{RC}{\geq \pi }_{r}^{RM},\;{\text{p}}_{\text{1}}^{\text{RC}}\leq {\text{p}}_{\text{1}}^{\text{RM}}$.

The proof of Theorem 6 is enclosed in the Appendix.

Theorem 6 illustrates that when retailers are core enterprises, the market demand, product green level, manufacturers’ profits and retailers’ profits for green product R&D jointly conducted by manufacturers and retailers are greater than the optimal value for manufacturers to conduct green product R&D, and the retail price of green products is lower than the retail price for manufacturers to conduct green product R&D.

**Theorem 7**. When 2αη − γ^2^ ≥ 0, $D^{RM}\geq D^{RMT},\;s_{m}^{RM}\geq s_{t}^{RMT},\;\pi_{m}^{RM}\geq \pi_{m}^{RMT},\;\pi_{r}^{RM}\geq \pi_{r}^{RMT}$. When αη − γ^2^ ≥ 0, ${\text{p}}_{\text{1}}^{\text{RM}}\leq {\text{p}}_{\text{1}}^{\text{RMT}}$.

The proof of Theorem 7 is enclosed in the Appendix.

Theorem 7 states that when retailers are core enterprises, the market demand, product green level, manufacturers’ profits and retailers’ profits for green product R&D by manufacturers are greater than the optimal value for manufacturers to outsource green product R&D to third-party companies, and the retail price of green products is lower than the retail price for manufacturers to outsource green product R&D to third-party companies.

**Theorem 8**. When 2αη − γ^2^ ≥ 0, $D^{RR}\geq D^{RRT},\;s_{m}^{RR}\geq s_{t}^{RRT},\;\pi_{m}^{RR}\geq \pi_{m}^{RRT},\pi_{r}^{RR}\geq \pi_{r}^{RRT}$. When αη − γ^2^ ≥ 0, ${\text{p}}_{\text{1}}^{\text{RR}}\leq {\text{p}}_{\text{1}}^{\text{RRT}}$.

The proof of Theorem 8 is enclosed in the Appendix.

Theorem 8 shows that when retailers are core enterprises, the market demand, product green level, manufacturers’ profits and retailers’ profits for green product R&D by retailers are greater than the optimal value for retailers to outsource green product R&D to third-party companies, and the retail price of green products is lower than the retail price for retailers to outsource green product R&D to third-party companies.

From Theorems 5–8, it can be seen that when retailers are core enterprises, manufacturers’ green product R&D is better than that of retailers. Manufacturers and retailers’ joint green product R&D is better than that of manufacturers or retailers. Manufacturers’ green product R&D is better than manufacturers’ outsourcing of green product R&D to third-party companies, Retailers’ green product R&D is better than retailers’ outsourcing of green product R&D to third-party companies.

### 4.3 Manufacturers and retailers have equal power

#### 4.3.1 Manufacturers conduct green product R&D (NM)

When manufacturers and retailers have equal power, they make decisions at the same time. Manufacturers determine the wholesale price and green level of products, and retailers determine the retail price of products.

Let p_1_ = w_1_ + U_1_, and from $\frac{\partial \pi_{r}}{\partial p_{1}}=0$, $p_{1}^{NM}=\frac{D_{0}+\alpha w_{1}+\gamma {\text{s}}_{m}}{2\alpha }$. From $\frac{\partial \pi_{m}}{\partial w_{1}}=0$, $\frac{\partial \pi_{m}}{\partial s_{m}}=0$, $w_{1}^{NM}=\frac{D_{0}+\alpha c_{1}-\alpha p_{1}+\gamma {\text{s}}_{m}}{\alpha }$, $s_{m}^{NM}=\frac{\left(w_{1}-c_{1}\right)\gamma }{\eta }$, simultaneous solution can be obtained:$w_{1}^{NM}=\frac{D_{0}\eta +\left(2\alpha \eta -\gamma^{2}\right)c_{1}}{3\alpha \eta -\gamma^{2}}$, $s_{m}^{NM}=\frac{{(D}_{0}-\alpha c_{1})\gamma }{3\alpha \eta -\gamma^{2}}$, $p_{1}^{NM}=\frac{{2D}_{0}\eta +\left(\alpha \eta -\gamma^{2}\right)c_{1}}{3\alpha \eta -\gamma^{2}}$. Substitute and $w_{1}^{NM}$, $s_{m}^{NM}$ and $p_{1}^{NM}$, then $D^{NM}=\frac{{(D}_{0}-\alpha c_{1})\alpha \eta }{3\alpha \eta -\gamma^{2}}$, $\pi_{m}^{NM}=\frac{{{(D}_{0}-\alpha c_{1})}^{2}(2\alpha \eta -\gamma^{2})\eta }{2{\left(3\alpha \eta -\gamma^{2}\right)}^{2}}$, $\pi_{r}^{NM}=\frac{{{(D}_{0}-\alpha c_{1})}^{2}\alpha \eta^{2}}{{\left(3\alpha \eta -\gamma^{2}\right)}^{2}}$, $\pi_{sc}^{NM}=\frac{{{(D}_{0}-\alpha c_{1})}^{2}\left(4\alpha \eta -\gamma^{2}\right)\alpha \eta^{2}}{{2(3\alpha \eta -\gamma^{2})}^{2}}$.

#### 4.3.2 Retailers conduct green product R&D (NR)

When retailers conduct green product R&D, they make decisions at the same time. Retailers determine the retail price and green level of products, and manufacturers determine the wholesale price of products.

Let p_1_ = w_1_ + U_1_, we can get $w_{1}^{NR}=\frac{D_{0}-\alpha p_{1}+\alpha c_{1}+\gamma s_{r}}{\alpha }$ by $\frac{\partial \pi_{m}}{\partial w_{1}}=0$, From $\frac{\partial \pi_{r}}{\partial p_{1}}=0$, $\frac{\partial \pi_{r}}{\partial s_{r}}=0$, $p_{1}^{NR}=\frac{D_{0}+\alpha w_{1}+\gamma s_{r}}{2\alpha }$, $s_{r}^{NR}=\frac{(p_{1}-w_{1})\;\gamma }{\eta }$. The simultaneous solutions show that $s_{r}^{NR}=\frac{(D_{0}-\alpha c_{1})\gamma }{3\alpha \eta -\gamma^{2}}$, $w_{1}^{NR}=\frac{D_{0}\eta +(2\alpha \eta -\gamma^{2})c_{1}}{3\alpha \eta -\gamma^{2}}$, $p_{1}^{NR}=\frac{{2D}_{0}\eta +\left(\alpha \eta -\gamma^{2}\right)c_{1}}{3\alpha \eta -\gamma^{2}}$. We can obtain $D^{NR}=\frac{{(D}_{0}-\alpha c_{1})\alpha \eta }{3\alpha \eta -\gamma^{2}}$, $\pi_{m}^{NR}=\frac{{{(D}_{0}-\alpha c_{1})}^{2}\alpha \eta^{2}}{{\left(3\alpha \eta -\gamma^{2}\right)}^{2}}$, $\pi_{r}^{NR}=\frac{{{(D}_{0}-\alpha c_{1})}^{2}\left(2\alpha \eta -\gamma^{2}\right)\alpha \eta^{2}}{2{\left(3\alpha \eta -\gamma^{2}\right)}^{2}}$, $\pi_{sc}^{NR}=\frac{{{(D}_{0}-\alpha c_{1})}^{2}\left(4\alpha \eta -\gamma^{2}\right)\alpha \eta^{2}}{{2(3\alpha \eta -\gamma^{2})}^{2}}$.

#### 4.3.3 Manufacturers outsource green product R&D to third-party enterprises (NMT)

When manufacturers outsource green product R&D to third-party enterprises, because manufacturers and retailers have equal power, they make decisions at the same time. Manufacturers decide the wholesale price w_1_, retailers decide the retail price, and third-party companies decide the wholesale price w_2_ and green level of products.

Assuming p_1_ = w_1_ + w_2_ + U_1_, according to the inverse induction method, from $\frac{\partial \pi_{t}}{\partial w_{2}}=0$, $\frac{\partial \pi_{t}}{\partial s_{t}}=0$, $w_{2}^{NMT}=\frac{D_{0}-\alpha p_{1}+\gamma s_{t}}{\alpha }$, $s_{t}^{NMT}=\frac{w_{2}\gamma }{\eta }$. From $\frac{\partial \pi_{r}}{\partial p_{1}}=0$, we can get $p_{1}^{NMT}=\frac{D_{0}+\gamma s_{t}+\alpha w_{1}}{2\alpha }$. From $\frac{\partial \pi_{m}}{\partial w_{1}}=0$, $w_{1}^{NMT}=\frac{D_{0}-\alpha p_{1}+\gamma s_{t}+\alpha w_{2}+\alpha c_{1}}{\alpha }$, By simultaneous solution, we can obtain $w_{1}^{NMT}=\frac{{2\eta D}_{0}+(2\alpha \eta -\gamma^{2})c_{1}}{4\alpha \eta -\gamma^{2}}$, $p_{1}^{NMT}=\frac{{3\eta D}_{0}+(\alpha \eta -\gamma^{2})c_{1}}{4\alpha \eta -\gamma^{2}}$, $w_{2}^{NMT}=\frac{{(D}_{0}-\alpha c_{1})\eta }{4\alpha \eta -\gamma^{2}}$, $s_{t}^{NMT}=\frac{{(D}_{0}-\alpha c_{1})\gamma }{4\alpha \eta -\gamma^{2}}$, then $D^{NMT}=\frac{{(D}_{0}-\alpha c_{1})\alpha \eta }{4\alpha \eta -\gamma^{2}}$, $\pi_{m}^{NMT}=\frac{{{(D}_{0}-\alpha c_{1})}^{2}\alpha \eta^{2}}{{\left(4\alpha \eta -\gamma^{2}\right)}^{2}}$, $\pi_{r}^{NMT}=\frac{{{(D}_{0}-\alpha c_{1})}^{2}\alpha \eta^{2}}{{\left(4\alpha \eta -\gamma^{2}\right)}^{2}}$, $\pi_{t}^{NMT}=\frac{{{\left(2\alpha \eta -\gamma^{2}\right)(D}_{0}-\alpha c_{1})}^{2}\eta }{2{\left(4\alpha \eta -\gamma^{2}\right)}^{2}}$, $\pi_{sc}^{NMT}=\frac{{{\left(10\alpha \eta -3\gamma^{2}\right)(D}_{0}-\alpha c_{1})}^{2}\eta }{2{\left(4\alpha \eta -\gamma^{2}\right)}^{2}}$.

#### 4.3.4 Retailers outsource green product R&D to third-party enterprises (NRT)

When retailers outsource green product R&D to third-party enterprises, because manufacturers and retailers have equal power, they make decisions at the same time. Manufacturers decide the wholesale price w_1_, retailers decide the retail price, and third-party companies decide the wholesale price w_2_ and green level of products.

Assuming p_1_ = w_1_ + w_2_ + U_1_, according to the inverse induction method, from $\frac{\partial \pi_{t}}{\partial w_{2}}=0$, $\frac{\partial \pi_{t}}{\partial s_{t}}=0$, ${\;w}_{2}^{NMT}=\frac{D_{0}-\alpha p_{1}+\gamma s_{t}}{\alpha }$, $s_{t}^{NMT}=\frac{w_{2}\gamma }{\eta }$. From $\frac{\partial \pi_{r}}{\partial p_{1}}=0$, $p_{1}^{NRT}=\frac{D_{0}+\gamma s_{t}+\alpha w_{1}+\alpha w_{2}}{2\alpha }$. From $\frac{\partial \pi_{m}}{\partial w_{1}}=0$, $w_{1}^{NRT}=\frac{D_{0}-\alpha p_{1}+\gamma s_{t}+\alpha c_{1}}{\alpha }$. By simultaneous solution, we can obtain ${\;w}_{1}^{NRT}=\frac{{\eta D}_{0}+(3\alpha \eta -\gamma^{2})c_{1}}{4\alpha \eta -\gamma^{2}}$, $p_{1}^{NRT}=\frac{{3\eta D}_{0}+(\alpha \eta -\gamma^{2})c_{1}}{4\alpha \eta -\gamma^{2}}$, $w_{2}^{NRT}=\frac{{(D}_{0}-\alpha c_{1})\eta }{4\alpha \eta -\gamma^{2}}$, $s_{t}^{NRT}=\frac{{(D}_{0}-\alpha c_{1})\gamma }{4\alpha \eta -\gamma^{2}}$, then $D^{NRT}=\frac{{(D}_{0}-\alpha c_{1})\alpha \eta }{4\alpha \eta -\gamma^{2}}$, $\pi_{m}^{NRT}=\frac{{{(D}_{0}-\alpha c_{1})}^{2}\alpha \eta^{2}}{{\left(4\alpha \eta -\gamma^{2}\right)}^{2}}$, $\pi_{r}^{NRT}=\frac{{{(D}_{0}-\alpha c_{1})}^{2}\alpha \eta^{2}}{{\left(4\alpha \eta -\gamma^{2}\right)}^{2}}$, $\pi_{t}^{NRT}=\frac{{{\left(2\alpha \eta -\gamma^{2}\right)(D}_{0}-\alpha c_{1})}^{2}\eta }{2{\left(4\alpha \eta -\gamma^{2}\right)}^{2}}$, $\pi_{sc}^{NRT}=\frac{{{\left(10\alpha \eta -3\gamma^{2}\right)(D}_{0}-\alpha c_{1})}^{2}\eta }{2{\left(4\alpha \eta -\gamma^{2}\right)}^{2}}$.

#### 4.3.5 Manufacturers and retailers jointly conduct green product R&D (NC)

When manufacturers and retailers jointly conduct green product R&D, because manufacturers and retailers have equal power, manufacturers and retailers make decisions at the same time, and manufacturers decide the wholesale price and green level of products. Retailers determine the retail price of products and the green level of products.

From $\frac{\partial \pi_{m}}{\partial w_{1}}=0$, $\frac{\partial \pi_{m}}{\partial s_{m}}=0,$ $w_{1}^{NC}=\frac{D_{0}-\alpha p_{1}+\gamma s_{m}+\gamma s_{r}+\alpha c_{1}}{\alpha }$, $s_{m}^{NC}=\frac{\left(w_{1}-c_{1}\right)\gamma }{\eta }$, simultaneous solution: $w_{1}^{NC}=\frac{D_{0}\eta -\alpha p_{1}\eta +\gamma s_{r}\eta +c_{1}(\alpha \eta -\gamma^{2})}{\alpha \eta -\gamma^{2}}$, $s_{m}^{NC}=\frac{\left(D_{0}-\alpha p_{1}+\gamma s_{r}\right)\gamma }{\alpha \eta -\gamma^{2}}$. From $\frac{\partial \pi_{r}}{\partial p_{1}}=0$, $\frac{\partial \pi_{r}}{\partial s_{r}}=0$, $p_{1}^{NC}=\frac{D_{0}+\alpha w_{1}+\gamma s_{m}+\gamma s_{r}}{2\alpha }$, $s_{r}^{NC}=\frac{(p_{1}-w_{1})\gamma }{\eta }$, simultaneous solution: ${\text{p}}_{\text{1}}^{\text{NC}}=\frac{D_{0}\eta +(\alpha \eta -\gamma^{2}{)w}_{1}+\gamma s_{m}\eta }{\text{2}\alpha \eta -\gamma^{2}}$, $s_{r}^{NC}=\frac{\left(D_{0}-\alpha w_{1}+\gamma s_{m}\right)\gamma }{\text{2}\alpha \eta -\gamma^{2}}$. Then $w_{1}^{NC}=\frac{D_{0}\eta +2c_{1}(\alpha \eta -\gamma^{2})}{3\alpha \eta -2\gamma^{2}}$, $s_{m}^{NC}=\frac{\left(D_{0}-\alpha c_{1}\right)\gamma }{3\alpha \eta -2\gamma^{2}}$, ${\text{p}}_{\text{1}}^{\text{NC}}=\frac{2D_{0}\eta +(\alpha \eta -2\gamma^{2})c_{1}}{\text{3}\alpha \eta -2\gamma^{2}}$, $s_{r}^{NC}=\frac{\left(D_{0}-\alpha c_{1}\right)\gamma }{3\alpha \eta -2\gamma^{2}}$, $D^{NC}=\frac{{(D}_{0}-\alpha c_{1})\alpha \eta }{3\alpha \eta -2\gamma^{2}}$, $\pi_{m}^{NC}=\frac{{{(D}_{0}-\alpha c_{1})}^{2}(2\alpha \eta -\gamma^{2})\eta }{2{\left(3\alpha \eta -2\gamma^{2}\right)}^{2}}$, $\pi_{r}^{NC}=\frac{{{(D}_{0}-\alpha c_{1})}^{2}(2\alpha \eta -\gamma^{2})\eta }{2{\left(3\alpha \eta -2\gamma^{2}\right)}^{2}}$, $\pi_{sc}^{NC}=\frac{{{(D}_{0}-\alpha c_{1})}^{2}(2\alpha \eta -\gamma^{2})\eta }{{\left(3\alpha \eta -2\gamma^{2}\right)}^{2}}$.

**Theorem 9**. When 3αη − 2γ^2^ > 0, D^NC^ ≥ D^NR^ = D^NM^, $s_{r}^{NC}\geq s_{r}^{NR}=s_{m}^{NM}{,\;\pi }_{m}^{NC}{\geq \pi }_{m}^{NR}\geq \pi_{m}^{NM},{\pi_{r}^{NC}\geq \pi }_{r}^{NM}{\geq \pi }_{r}^{NR},{\text{p}}_{\text{1}}^{\text{NC}}\geq {\text{p}}_{\text{1}}^{\text{NM}}={\text{p}}_{\text{1}}^{\text{NR}}$.

The proof of Theorem 9 is enclosed in the Appendix.

Theorem 9 shows that when manufacturers and retailers have equal power, the market demand, product green level, product retail price, manufacturers’ profits, and retailers’ profits of green product R&D jointly conducted by manufacturers and retailers are greater than the optimal value for manufacturers or retailers to conduct green product R&D.

**Theorem 10**. When 3αη − γ^2^ > 0, $D^{NM}\geq D^{NMT},\;s_{m}^{NM}\geq s_{t}^{NMT},\pi_{r}^{NM}\geq \pi_{r}^{NMT}$. When αη − γ^2^ ≥ 0, $\pi_{m}^{NM}\geq \pi_{m}^{NMT},{\text{p}}_{\text{1}}^{\text{NM}}\leq {\text{p}}_{\text{1}}^{\text{NMT}}$.

The proof of Theorem 10 is enclosed in the Appendix.

Theorem 10 states that when manufacturers and retailers have equal power, the market demand, product green level, manufacturers’ profits and retailers’ profits for green product R&D by manufacturers are greater than the optimal value for manufacturers outsourcing green product R&D to third-party companies, and the retail price of green products is lower than the retail price for manufacturers outsourcing green product R&D to third-party companies.

**Theorem 11**. When 3αη − γ^2^ ≥ 0, $D^{NR}\geq D^{NRT},\;s_{m}^{NR}\geq s_{t}^{NRT},\;\pi_{m}^{NR}\geq \pi_{m}^{NRT},{\text{p}}_{\text{1}}^{\text{NR}}\leq {\text{p}}_{\text{1}}^{\text{NRT}}$.

The proof of Theorem 11 is enclosed in the Appendix.

Theorem 11 shows that when manufacturers and retailers have equal power, the market demand, product green level, manufacturers’ profits and retailers’ profits for green product R&D by retailers are greater than the optimal value for retailers to outsource green product R&D to third-party companies, and the retail price of green products is lower than the retail price for retailers to outsource green product R&D to third-party companies.

From Theorems 9–11, when manufacturers and retailers have equal power, it is better for manufacturers and retailers to jointly carry out green product R&D than that of manufacturers or retailers, and it is better for manufacturers to carry out green product R&D than for manufacturers outsourcing green product R&D to third-party companies, and it is better for retailers outsourcing green product R&D to third-party companies.

## 5. Equilibrium results analysis in different power structures

**Theorem 12**. When manufacturers conduct green product R&D, If αη − γ^2^ ≥ 0, then ${\text{D}}^{\text{NM}}\geq D^{RM}\geq D^{MM}$. When retailers conduct green product R&D, If αη − γ^2^ ≥ 0, D^NR^ ≥ D^MR^ ≥ D^RR^. When manufacturers and retailers jointly conduct green product R&D, if αη − γ^2^ ≥ 0, then D^NC^ ≥ D^MC^ = D^RC^. When manufacturers outsource green product R&D to third-party companies, if 2αη − γ^2^ ≥ 0, D^NMT^ ≥ D^RMT^ = D^MMT^. When retailers outsource green product R&D to third-party companies, if 2αη − γ^2^ ≥ 0, D^NRT^ ≥ D^RRT^ = D^MRT^.

The proof of Theorem 12 is enclosed in the Appendix.

**Theorem 13**. When manufacturers conduct green product R&D, if αη − γ^2^ ≥ 0, $s_{m}^{NM}\geq s_{m}^{RM}\geq s_{m}^{MM}$. When retailers conduct green product R&D, if αη − γ^2^ ≥ 0, $s_{r}^{NR}\geq s_{r}^{MR}\geq s_{r}^{RR}.\;$ When manufacturers and retailers jointly conduct green product R&D, if αη − γ^2^ ≥ 0, $s_{m}^{NC}\geq s_{m}^{MC}=s_{m}^{RC}$. When manufacturers outsource green product R&D to third-party companies, if 2αη − γ^2^ ≥ 0, $s_{t}^{NMT}\geq s_{t}^{MMT}=s_{t}^{RMT}$. When retailers outsource green product R&D to third-party companies, if 2αη − γ^2^ ≥ 0, $s_{t}^{NRT}\geq s_{t}^{RRT}=s_{t}^{MRT}$.

The proof of Theorem 13 is enclosed in the Appendix.

Theorems 12–13 illustrate that no matter who conducts green product R&D, when manufacturers and retailers have equal power, the market demand and green level of green products are the highest.

**Theorem 14**. When manufacturers conduct green product R&D, if αη − γ^2^ ≥ 0, $p_{1}^{MM}\geq {\text{p}}_{\text{1}}^{\text{RM}}\geq p_{1}^{NM}$. When retailers conduct green product R&D, if αη − γ^2^ ≥ 0, $p_{1}^{RR}\geq {\text{p}}_{\text{1}}^{\text{MR}}\geq p_{1}^{NR}.\;$ When manufacturers and retailers jointly conduct green product R&D, if αη − γ^2^ ≥ 0, ${\text{p}}_{\text{1}}^{\text{MC}}={\text{p}}_{\text{1}}^{\text{RC}}\geq {\text{p}}_{\text{1}}^{\text{NC}}$. When manufacturers outsource green product R&D to third-party companies, if 2αη − γ^2^ ≥ 0, $p_{1}^{MMT}=p_{1}^{RMT}\geq p_{1}^{NMT}$. When retailers outsource green product R&D to third-party companies, if 2αη − γ^2^ ≥ 0, $p_{1}^{MRT}=p_{1}^{RRT}\geq p_{1}^{NRT}$.

The proof of Theorem 14 is enclosed in the Appendix.

Theorem 14 shows that when retailers conduct green product R&D, the retail price with retailers as core enterprises is the highest, while in other green product R&D modes, the retail price with manufacturers as core enterprises is the highest. No matter who conducts green product R&D, when manufacturers and retailers have equal power, the retail price is the lowest.

**Theorem 15**. When manufacturers conduct green product R&D, if αη − γ^2^ ≥ 0, $\pi_{m}^{MM}\geq \pi_{m}^{NM}\;\geq \pi_{m}^{RM},\pi_{r}^{RM}\geq \pi_{r}^{NM}\geq \pi_{r}^{MM}$. When retailers carry out green product R&D, if αη − γ^2^ ≥ 0, $\pi_{m}^{MR}\geq \pi_{m}^{NR}\geq \pi_{m}^{RR},\pi_{r}^{RR}\geq \pi_{r}^{NR}\geq \pi_{r}^{MR}$. When manufacturers and retailers jointly carry out green product R&D, if αη − γ^2^ ≥ 0, $\pi_{m}^{MC}\geq \pi_{m}^{NC}\geq \pi_{m}^{RC},\pi_{r}^{RC}\geq \pi_{r}^{NC}\geq \pi_{r}^{MC}$. When manufacturers outsource green product R&D to third-party companies, if 2αη − γ^2^ ≥ 0, $\pi_{m}^{MMT}\geq \pi_{m}^{NMT}\geq \pi_{m}^{RMT},\pi_{r}^{RMT}\geq \pi_{r}^{NMT}\geq \pi_{r}^{MMT}$. When retailers outsource green product R&D to third-party companies, if 2αη − γ^2^ ≥ 0, $\pi_{m}^{MRT}\geq \pi_{m}^{NRT}\geq \pi_{m}^{RRT},\pi_{r}^{RRT}\geq \pi_{r}^{NRT}\geq \pi_{r}^{MRT}$.

The proof of Theorem 15 is enclosed in the Appendix.

Theorem 15 shows that no matter who conducts green product R&D, when manufacturers are core enterprises, manufacturers’ profits are the highest, and when retailers are core enterprises, retailers’ profits are the highest.

## 6 Supply chain coordination of two-part pricing contract

### 6.1 Centralized decision-making when manufacturers or retailers conduct green product R&D

In centralized decision-making, manufacturers and retailers jointly pursue the maximization of the overall profit of the supply chain, the total profit of the supply chain is:

$$\pi_{sc}=\left(p_{1}-c_{1}\right){(D}_{0}-\alpha p_{1}+\gamma s_{i})-\frac{1}{2}\eta s_{i}^{2}$$ (13)

Let $\frac{\partial \pi_{sc}}{\partial p_{1}}=0$, $\frac{\partial \pi_{sc}}{\partial s_{i}}=0$, we can obtain $p_{1}^{J}=\frac{D_{0}+\alpha c_{1}+\gamma s}{2\alpha }$, $s^{J}=\frac{\left(p_{1}-c_{1}\right)\gamma }{\eta }$, The simultaneous solutions are $p_{1}^{J}=\frac{D_{0}\eta +(\alpha \eta -\gamma^{2})c_{1}}{2\alpha \eta -\gamma^{2}}$, $s_{i}^{J}=\frac{{(D}_{0}-\alpha c_{1})\gamma }{2\alpha \eta -\gamma^{2}}$. By substituting $p_{1}^{J}$ and $s_{i}^{J}$ into π_sc_, the optimal total revenue of the supply chain can be obtained $\pi_{sc}^{J}=\frac{{{(D}_{0}-\alpha c_{1})}^{2}\eta }{2(2\alpha \eta -\gamma^{2})}$. Comparing with decentralized decision, the difference between the optimal total revenue of supply chain is:$\Delta \pi =\frac{{{(D}_{0}-\alpha c_{1})}^{2}\eta }{2(2\alpha \eta -\gamma^{2})}-\frac{{{3(D}_{0}-\alpha c_{1})}^{2}\eta }{8\left(2\alpha \eta -\gamma^{2}\right)}=\frac{{{(D}_{0}-\alpha c_{1})}^{2}\eta }{8{\left(2\alpha \eta -\gamma^{2}\right)}^{2}}\geq 0$, which indicates that the optimal result in decentralized decision-making does not reach supply chain coordination.

### 6.2 Supply chain coordination when manufacturers or retailers conduct green product R&D

From Theorems 1 and 5, it can be seen that when manufacturers or retailers conduct green product R&D, it is optimal for retailers to conduct green product R&D when the manufacturer is the core enterprise, and it is optimal for manufacturers to conduct green product R&D when the retailer is the core enterprise. In order to maximize the total revenue of the supply chain and achieve the win-win goal of manufacturers and retailers, this paper uses two-part pricing contracts to coordinate the optimal modes.

Assume that the two-part pricing contracts is: Z = F + w_1_ D, where F is the fixed fee, w_1_ is the wholesale price of unit product, and D is the product demand. When manufacturers are core enterprises and retailers conduct green product R&D, manufacturers sell products to retailers and manufacturers charge retailers fixed fees F. Then the profits of manufacturers and retailers are:

$$\pi_{m}=\left(w_{1}-c_{1}\right){(D}_{0}-\alpha p_{1}+\gamma s_{r})+F$$ (14)

$$\pi_{r}=\left(p_{1}-w_{1}\right){(D}_{0}-\alpha p_{1}+\gamma s_{r})-\frac{1}{2}\eta s_{r}^{2}-F$$ (15)

According to the reverse induction method, retailers need to set the product price $p_{1}^{MR*}\;$ and the product green level $s_{r}^{MR*}$ as the optimal solution in centralized decision-making, that is, $p_{1}^{MR*}=p_{1}^{J}$, $s_{r}^{MR*}=s_{i}^{J}$, and substitute π_m_ to obtain the optimal value $w_{1}^{MR*}$.

From ${\text{p}}_{\text{1}}^{\text{MR}*}=\frac{D_{0}\eta +(\alpha \eta -\gamma^{2}{)w}_{1}}{\text{2}\alpha \eta -\gamma^{2}}=\frac{D_{0}\eta +(\alpha \eta -\gamma^{2})c_{1}}{2\alpha \eta -\gamma^{2}}$, It can be obtained $w_{1}^{MR*}=c_{1}$, and substitute $p_{1}^{MR*}$, $s_{r}^{MR*}$ and $w_{1}^{MR*}\;$ into $\pi_{m}^{MR*}$, $\pi_{r}^{MR*}$, it can be obtained $\pi_{m}^{MR*}=F$, $\pi_{r}^{MR*}=\frac{{{(D}_{0}-\alpha c_{1})}^{2}\eta }{2(2\alpha \eta -\gamma^{2})}-F$. To ensure $\pi_{m}^{MR*}\geq \pi_{m}^{MR}$, $\pi_{r}^{MR*}\geq \pi_{r}^{MR}$, we can obtain $\frac{{\left(D_{0}-\alpha c_{1}\right)}^{2}\eta }{4(2\alpha \eta -\gamma^{2})}\leq F\leq \frac{{{3(D}_{0}-\alpha c_{1})}^{2}\eta }{8\left(2\alpha \eta -\gamma^{2}\right)}$.

Therefore, coordination can be achieved by adopting two-part pricing contracts. The profits of both manufacturers and retailers increase.

When retailers are core enterprises and manufacturers conduct green product R&D, manufacturers sell products to retailers and manufacturers pay retailers fixed fees F. Then the profits of manufacturers and retailers are:

$$\pi_{m}=\left(w_{1}-c_{1}\right){(D}_{0}-\alpha p_{1}+\gamma s_{m})-\frac{1}{2}\eta s_{m}^{2}-F$$ (16)

$$\pi_{r}=\left(p_{1}-w_{1}\right){(D}_{0}-\alpha p_{1}+\gamma s_{m})+F$$ (17)

According to the reverse induction method, manufacturers set the product green level $s_{m}^{RM*}\;$ as the optimal solution in centralized decision-making, that is, $s_{m}^{RM*}=s_{i}^{J}$. At the same time, by determining the wholesale price $w_{1}^{RM*}$, the retailer’s optimal product price decision $p_{1}^{RM*}$ is the optimal value $p_{1}^{J}$ of the product price in centralized decision-making.

According to $s_{m}^{RM*}=\frac{(D_{0}-\alpha p_{1})\gamma }{\alpha \eta -\gamma^{2}}=\frac{{(D}_{0}-\alpha c_{1})\gamma }{2\alpha \eta -\gamma^{2}}$, we can get $p_{1}^{RM*}=p_{1}^{J}=\frac{D_{0}\eta +(\alpha \eta -\gamma^{2})c_{1}}{\left(2\alpha \eta -\gamma^{2}\right)}$. Substitute $p_{1}^{RM*}$ into $w_{1}^{RM*}=\frac{{(D}_{0}-\alpha p_{1})\eta }{\left(\alpha \eta -\gamma^{2}\right)}+c_{1}$, we can obtain $w_{1}^{RM*}=\frac{D_{0}\eta +(\alpha \eta -\gamma^{2})c_{1}}{2\alpha \eta -\gamma^{2}}$. Substitute $p_{1}^{RM*}$, $s_{m}^{RM*}$ and $w_{1}^{RM*}$ into $\pi_{m}^{RM*}$ and $\pi_{r}^{RM*}$ to get $\pi_{m}^{RM*}=\frac{{{(D}_{0}-\alpha c_{1})}^{2}\eta }{2(2\alpha \eta -\gamma^{2})}-F$, $\pi_{r}^{RM*}=F$. To ensure $\pi_{m}^{RM*}\geq \pi_{m}^{RM}$, $\pi_{r}^{RM*}\geq \pi_{r}^{RM}$, it is obtained that $\frac{{\left(D_{0}-\alpha c_{1}\right)}^{2}\eta }{4(2\alpha \eta -\gamma^{2})}\leq F\leq \frac{{{3(D}_{0}-\alpha c_{1})}^{2}\eta }{8\left(2\alpha \eta -\gamma^{2}\right)}$.

Therefore, when two-part pricing contracts are adopted for supply chain coordination, the profits of both manufacturers and retailers increase.

### 6.3 Centralized decision-making when manufacturers and retailers jointly conduct green product R&D

When manufacturers and retailers jointly carry out green product R&D, the total profit of the supply chain is:

$$\pi_{sc}=\left(p_{1}-c_{1}\right)\left(D_{0}-\alpha p_{1}+\gamma s_{m}+\gamma s_{r}\right)-\frac{1}{2}\eta s_{m}^{2}-\frac{1}{2}\eta s_{r}^{2}$$ (18)

From $\frac{\partial \pi_{sc}}{\partial p_{1}}=0$, $\frac{\partial \pi_{sc}}{\partial s_{m}}=0$, $\frac{\partial \pi_{sc}}{\partial s_{r}}=0$, we can obtain $p_{1}^{J}=\frac{D_{0}+\alpha c_{1}+\gamma s_{m}+\gamma s_{r}}{2\alpha }$, $s_{m}^{J}=s_{r}^{J}=\frac{\left(p_{1}-c_{1}\right)\gamma }{\eta }$, the simultaneous solutions are $p_{1}^{J}=\frac{D_{0}\eta +(\alpha \eta -2\gamma^{2})\;c_{1}}{2(\alpha \eta -\gamma^{2})\;}$, $s_{m}^{J}=s_{r}^{J}=\frac{{(D}_{0}-\alpha c_{1})\gamma \;}{2(\alpha \eta -\gamma^{2})}$. By substituting $p_{1}^{J}$, $s_{m}^{J}$ and $s_{r}^{J}$ into π_sc_, the optimal total revenue of the supply chain in centralized decision-making can be obtained $\pi_{sc}^{J}=\frac{{{(D}_{0}-\alpha c_{1})}^{2}\eta }{4(\alpha \eta -\gamma^{2})\;}$. From $\pi_{sc}^{J}\geq \pi_{sc}^{MC}=\pi_{sc}^{RC}$, we can obtain $\frac{{{(D}_{0}-\alpha c_{1})}^{2}\eta }{4(\alpha \eta -\gamma^{2})\;}\geq \frac{{{(D}_{0}-\alpha c_{1})}^{2}(3\alpha \eta -2\gamma^{2})\;\eta }{{\left(4\alpha \eta -3\gamma^{2}\;\right)}^{2}}$, simplified to (γ^2^ − 2αη)^2^ ≥ 0. From $\pi_{sc}^{J}\geq \pi_{sc}^{NC}$, we can obtain $\frac{{{(D}_{0}-\alpha c_{1})}^{2}\eta }{4(\alpha \eta -\gamma^{2})\;}\geq \frac{{{(D}_{0}-\alpha c_{1})}^{2}(2\alpha \eta -\gamma^{2})\;\eta }{{\left(3\alpha \eta -2\gamma^{2}\right)}^{2}}$, simplified to α^2^ η^2^ ≥ 0, which means that the optimal solution of decentralized decision-making in different power structures has not reached the coordination of supply chain.

### 6.4 Supply chain coordination when manufacturers and retailers jointly conduct green product R&D

From Theorems 2, 6 and 9, it can be seen that in the three power structures, it is the best choice for manufacturers and retailers to jointly carry out green product R&D, but they have not achieved supply chain coordination, so two-part pricing contracts are used to coordinate the supply chain.

According to two-part pricing contracts: Z = F + w_1_ D, when manufacturers are core enterprises, manufacturers and retailers jointly carry out green products R&D. Manufacturers sell products to retailers and manufacturers charge retailers a fixed fee F. Then the profits of manufacturers and retailers are:

$$\pi_{m}=\left(w_{1}-c_{1}\right){(D}_{0}-\alpha p_{1}+\gamma s_{m}+\gamma s_{r})-\frac{1}{2}\eta s_{m}^{2}+F$$ (19)

$$\pi_{r}=\left(p_{1}-w_{1}\right){(D}_{0}-\alpha p_{1}+\gamma s_{m}+\gamma s_{r})-\frac{1}{2}\eta s_{r}^{2}-F$$ (20)

According to the reverse induction method, to achieve the maximum benefit of supply chains in centralized decision-making, retailers need to set the product price $p_{1}^{MC*}$ and the product green level $s_{r}^{MC*}$ as the optimal solution in centralized decision-making, that is, $p_{1}^{MC*}=p_{1}^{J}$, $s_{r}^{MC*}=s_{r}^{J}$. Then manufacturers’ optimal decision $w_{1}^{MC*}$ and $s_{m}^{MC*}$ can be obtained.

From ${\text{p}}_{\text{1}}^{\text{MC}*}=\frac{D_{0}\eta +(\alpha \eta -\gamma^{2}\;{)w}_{1}+\gamma s_{m}\eta }{\text{2}\alpha \eta -\gamma^{2}}=\frac{D_{0}\eta +(\alpha \eta -2\gamma^{2})\;c_{1}}{2(\alpha \eta -\gamma^{2})}$, $s_{r}^{MC*}=\frac{\left(D_{0}-\alpha w_{1}+\gamma s_{m}\right)\gamma }{\text{2}\alpha \eta -\gamma^{2}}=\frac{{(D}_{0}-\alpha c_{1})\gamma \;}{2(\alpha \eta -\gamma^{2})\;}$, It can be obtained $w_{1}^{MC*}=c_{1}$, $s_{m}^{MC*}=\frac{{(D}_{0}-\alpha c_{1})\gamma \;}{2(\alpha \eta -\gamma^{2})\;}$. Substitute $p_{1}^{MC*}$, $s_{r}^{MC*}$, $s_{m}^{MC*}$ and $w_{1}^{MC*}$ to get $\pi_{m}^{MC*}=-\frac{{{(D}_{0}-\alpha c_{1})}^{2}\gamma^{2}\eta \;}{8{(\alpha \eta -\gamma^{2})\;}^{2}}+F$, $\pi_{r}^{MC*}=\frac{{{(D}_{0}-\alpha c_{1})}^{2}(2\alpha \eta -\gamma^{2})\eta }{8{(\alpha \eta -\gamma^{2})\;}^{2}}-F$. To ensure $\pi_{m}^{MC*}\geq \pi_{m}^{MC}$, $\pi_{r}^{MC*}\geq \pi_{r}^{MC}$, it is obtained $\frac{{\left(D_{0}-\alpha c_{1}\right)}^{2}{(2\alpha \eta -\gamma^{2})\;}^{2}\eta }{8{\left(\alpha \eta -\gamma^{2}\right)}^{2}(4\alpha \eta -3\gamma^{2})\;}\leq F\leq \frac{{{(D}_{0}-\alpha c_{1})}^{2}{(2\alpha \eta -\gamma^{2})\;}^{2}(6\alpha \eta -5\gamma^{2})\;\eta }{8{\left(\alpha \eta -\gamma^{2}\right)}^{2}{(4\alpha \eta -3\gamma^{2})\;}^{2}}$.

Therefore, two-part pricing contracts can realize the supply chain coordination when manufacturers are core enterprises. The profits of both manufacturers and retailers increase.

When retailers are core enterprises, manufacturers and retailers jointly carry out green products R&D. Manufacturers sell products to retailers and pay retailers a fixed fee F. Then the profits of manufacturers and retailers are:

$$\pi_{m}=\left(w_{1}-c_{1}\right){(D}_{0}-\alpha p_{1}+\gamma s_{m}+\gamma s_{r})-\frac{1}{2}\eta s_{m}^{2}-F$$ (21)

$$\pi_{r}=\left(p_{1}-w_{1}\right){(D}_{0}-\alpha p_{1}+\gamma s_{m}+\gamma s_{r})-\frac{1}{2}\eta s_{r}^{2}+F$$ (22)

According to the reverse induction method, to achieve the maximum benefit of the supply chain in centralized decision-making, manufacturers need to set the product green level $s_{m}^{RC*}$ equal to the optimal solution in centralized decision-making, that is, $s_{m}^{RC*}=s_{m}^{J}$. At the same time, by setting the wholesale price $w_{1}^{RC*}$, the retailer’s optimal product prices $p_{1}^{RC*}$ and $s_{r}^{RC*}$ are equal to the optimal solution in centralized decision-making.

According to $s_{m}^{RC}=\frac{\left(D_{0}-\alpha p_{1}+\gamma s_{r}\right)\gamma }{\left(\alpha \eta -\gamma^{2}\right)}=\frac{{(D}_{0}-\alpha c_{1})\gamma }{2(\alpha \eta -\gamma^{2})}$, Substitution $s_{r}^{J}=\frac{{(D}_{0}-\alpha c_{1})\gamma }{2(\alpha \eta -\gamma^{2})}$ to get $p_{1}^{RC*}=\frac{D_{0}\eta +(\alpha \eta -2\gamma^{2})c_{1}}{2(\alpha \eta -\gamma^{2})}$. Substitute $p_{1}^{RC*}$ and $s_{r}^{RC*}$ into $w_{1}^{RC}=\frac{{(D}_{0}\eta -\alpha p_{1}\eta +\gamma s_{r}\eta +c_{1}(\alpha \eta -\gamma^{2})}{\alpha \eta -\gamma^{2}}$, we can get $w_{1}^{RC*}=\frac{D_{0}\eta +(\alpha \eta -2\gamma^{2})c_{1}}{2(\alpha \eta -\gamma^{2})}$. Substitute $p_{1}^{RC*}$, $s_{r}^{RC*}$, $s_{m}^{RC*}$ and $w_{1}^{RC*}$ into $\pi_{m}^{RC*}\;$ and $\pi_{r}^{RC*}$ to get $\pi_{m}^{RC*}=\frac{{{(D}_{0}-\alpha c_{1})}^{2}(2\alpha \eta -\gamma^{2})\eta }{8{\left(\alpha \eta -\gamma^{2}\right)}^{2}}-F$, $\pi_{r}^{RC*}=-\frac{{{(D}_{0}-\alpha c_{1})}^{2}\gamma^{2}\eta }{8{\left(\alpha \eta -\gamma^{2}\right)}^{2}}+F.\;$ To ensure $\pi_{m}^{RC*}\geq \pi_{m}^{RC}$, $\pi_{r}^{RC*}\geq \pi_{r}^{RC}$, it is obtained $\frac{{\left(D_{0}-\alpha c_{1}\right)}^{2}{\left(2\alpha \eta -\gamma^{2}\right)}^{2}\eta }{8{\left(\alpha \eta -\gamma^{2}\right)}^{2}(4\alpha \eta -3\gamma^{2})}\leq F\leq \frac{{{(D}_{0}-\alpha c_{1})}^{2}{\left(2\alpha \eta -\gamma^{2}\right)}^{2}(6\alpha \eta -5\gamma^{2})\eta }{8{\left(\alpha \eta -\gamma^{2}\right)}^{2}{\left(4\alpha \eta -3\gamma^{2}\right)}^{2}}$.

Therefore, two-part pricing contract can realize the supply chain coordination when retailers are core enterprises. The profits of both manufacturers and retailers increase, and the total profits of supply chain also reach the optimal total profits in centralized decision-making.

When the power of manufacturers and retailers is equal, manufacturers and retailers jointly carry out green products R&D, manufacturers wholesale products to retailers, and manufacturers charge retailers a fixed fee F. Then the profits of manufacturers and retailers are Formulas (21) and (22) respectively.

To achieve the maximum benefit of supply chain in centralized decision-making, retailers need to set the product price $p_{1}^{NC*}$ and product green level $s_{r}^{NC*}$ as the optimal solution in centralized decision-making, that is, $p_{1}^{NC*}=p_{1}^{J}$, $s_{r}^{NC*}=s_{r}^{J}$. Manufacturers need to set the product green level $s_{m}^{NC*}$ equal to the optimal solution in centralized decision-making, that is, $s_{m}^{NC*}=s_{m}^{J}$, to obtain the wholesale price $w_{1}^{NC*}$.

From $w_{1}^{NC*}=\frac{D_{0}\eta -\alpha p_{1}\eta +\gamma s_{r}\eta +c_{1}(\alpha \eta -\gamma^{2})}{\left(\alpha \eta -\gamma^{2}\right)}$, substituted ${\text{p}}_{\text{1}}^{J}=\frac{D_{0}\eta +(\alpha \eta -2\gamma^{2})c_{1}}{2(\alpha \eta -\gamma^{2})}$, $s_{r}^{J}=\frac{{(D}_{0}-\alpha c_{1})\gamma }{2(\alpha \eta -\gamma^{2})}\;$ into $w_{1}^{NC*}$, we obtain $w_{1}^{NC*}=\frac{D_{0}\eta +(\alpha \eta -2\gamma^{2})c_{1}}{2(\alpha \eta -\gamma^{2})}$, ${\;s}_{m}^{NC*}=\frac{{(D}_{0}-\alpha c_{1})\gamma }{2(\alpha \eta -\gamma^{2})}$. Substitute $p_{1}^{NC*}$, $s_{r}^{NC*}$, $s_{m}^{NC*}$ and $w_{1}^{NC*}$ into $\pi_{m}^{NC}\;$ and $\pi_{r}^{NC}$, we obtain $\pi_{m}^{NC*}=\frac{{{(D}_{0}-\alpha c_{1})}^{2}(2\alpha \eta -\gamma^{2})\eta }{8{\left(\alpha \eta -\gamma^{2}\right)}^{2}}-F$, $\pi_{r}^{NC*}=-\frac{{{(D}_{0}-\alpha c_{1})}^{2}\gamma^{2}\eta }{8{\left(\alpha \eta -\gamma^{2}\right)}^{2}}+F$. To ensure $\pi_{m}^{NC*}\geq \pi_{m}^{NC}$, $\pi_{r}^{NC*}\geq \pi_{r}^{NC}$, it is obtained $\frac{{\left(D_{0}-\alpha c_{1}\right)}^{2}(4\gamma^{4}-11\gamma^{2}\alpha \eta +8\alpha^{2}\eta^{2})\alpha \eta^{2}}{8{\left(\alpha \eta -\gamma^{2}\right)}^{2}{\left(3\alpha \eta -2\gamma^{2}\right)}^{2}}\leq F\leq \frac{{{(D}_{0}-\alpha c_{1})}^{2}(2\alpha \eta -\gamma^{2})(5\alpha \eta -4\gamma^{2})\alpha \eta^{2}}{8{\left(\alpha \eta -\gamma^{2}\right)}^{2}{\left(3\alpha \eta -2\gamma^{2}\right)}^{2}}$.

Therefore, the adoption of two-part pricing contracts can realize the supply chain coordination when the power of manufacturers and retailers is equal. The revenues of both manufacturers and retailers increase, and the total revenue of the supply chain also reaches the optimal total revenue in centralized decision-making.

## 7. The numerical simulation analysis

According to the meaning and value range of each variable in the model, we set α = 0.1, c_1_ = 10, D_0_ = 500, γ = 0.2. The numerical simulation results are shown in the following figures.

**Fig 1:**
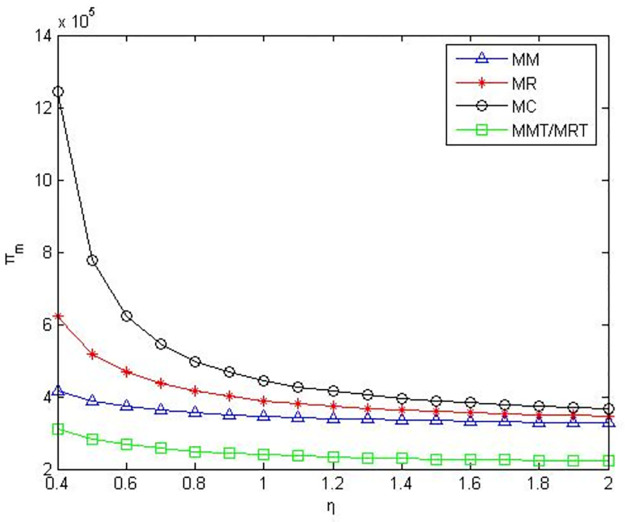
Manufacturers’ profits in the five R&D modes when manufacturers as core enterprises.

**Fig 2:**
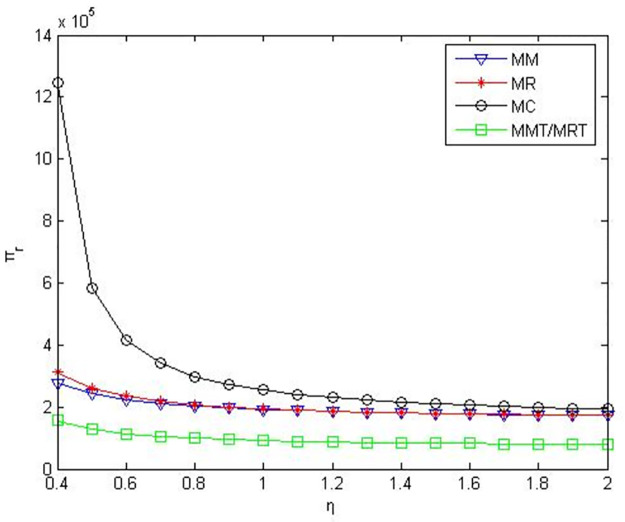
Retailers’ profits in the five R&D modes when manufacturers as core enterprises.

**Fig 3:**
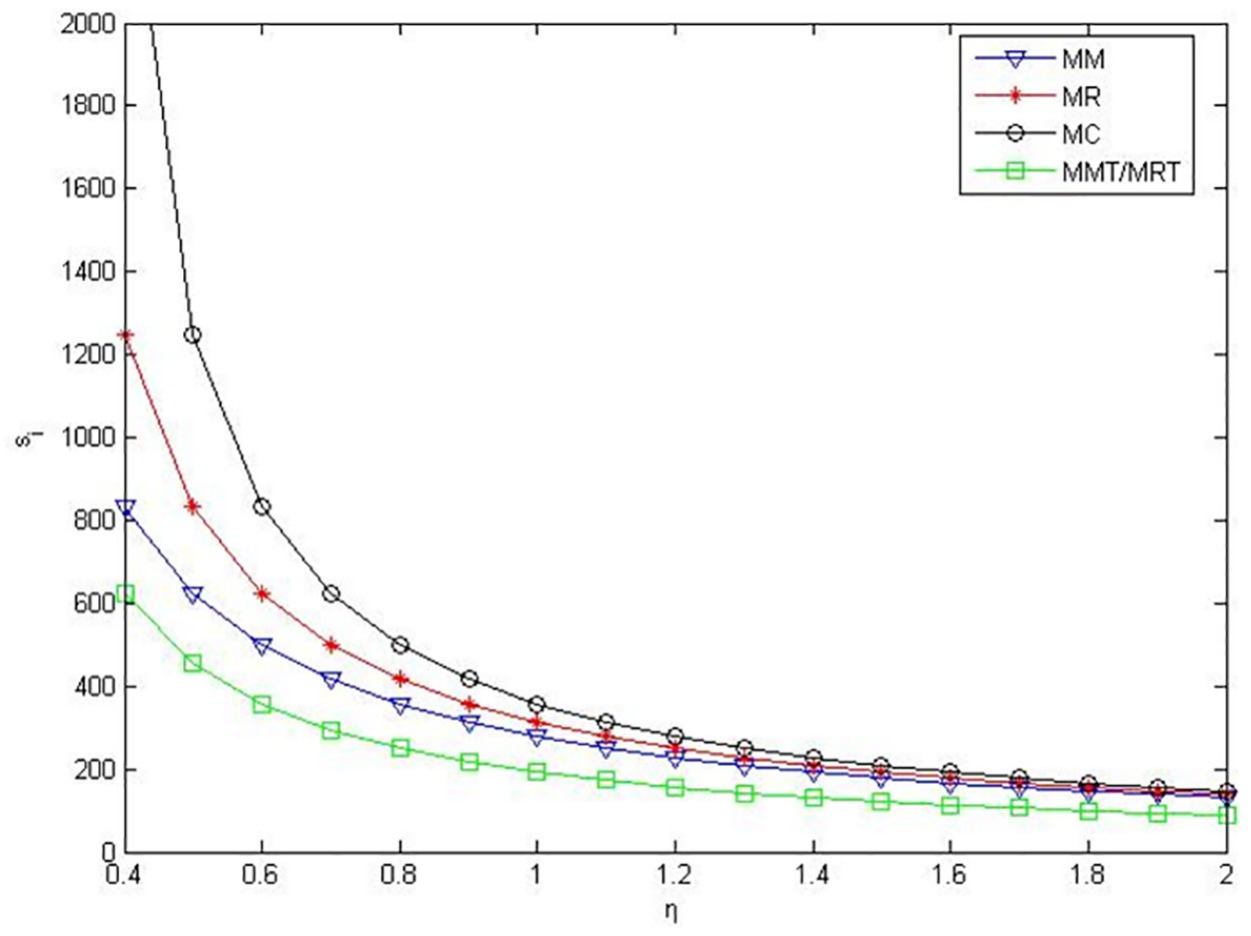
Product green levels in the five R&D modes when manufacturers as core enterprises.

As can be seen from Figs 1–3, when manufacturers are core enterprises, compared to manufacturers’ green product R&D, manufacturers’ profits, retailers’ profits, and product green level are greater when retailers conduct green product R&D. Compared to manufacturers or retailers’ green product R&D, when manufacturers and retailers jointly conduct green product R&D, manufacturers’ profits, retailers’ profits, and product green level are higher. Compared to manufacturers outsourcing green product R&D to third-party companies, manufacturers’ profits, retailers’ profits and product green level are greater when manufacturers conduct green product R&D. Compared to retailers outsourcing green product R&D to third-party companies, manufacturers’ profits, retailers’ profits and product green level are greater when retailers conduct green product R&D.

**Fig 4:**
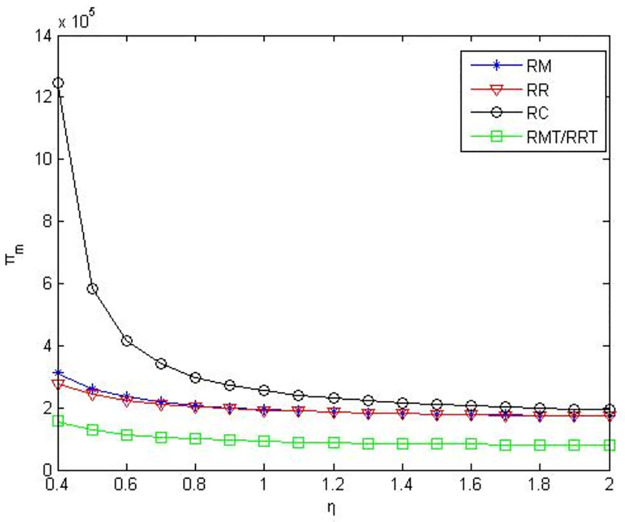
Manufacturers’ profits in the five R&D modes when retailers as core enterprises.

**Fig 5:**
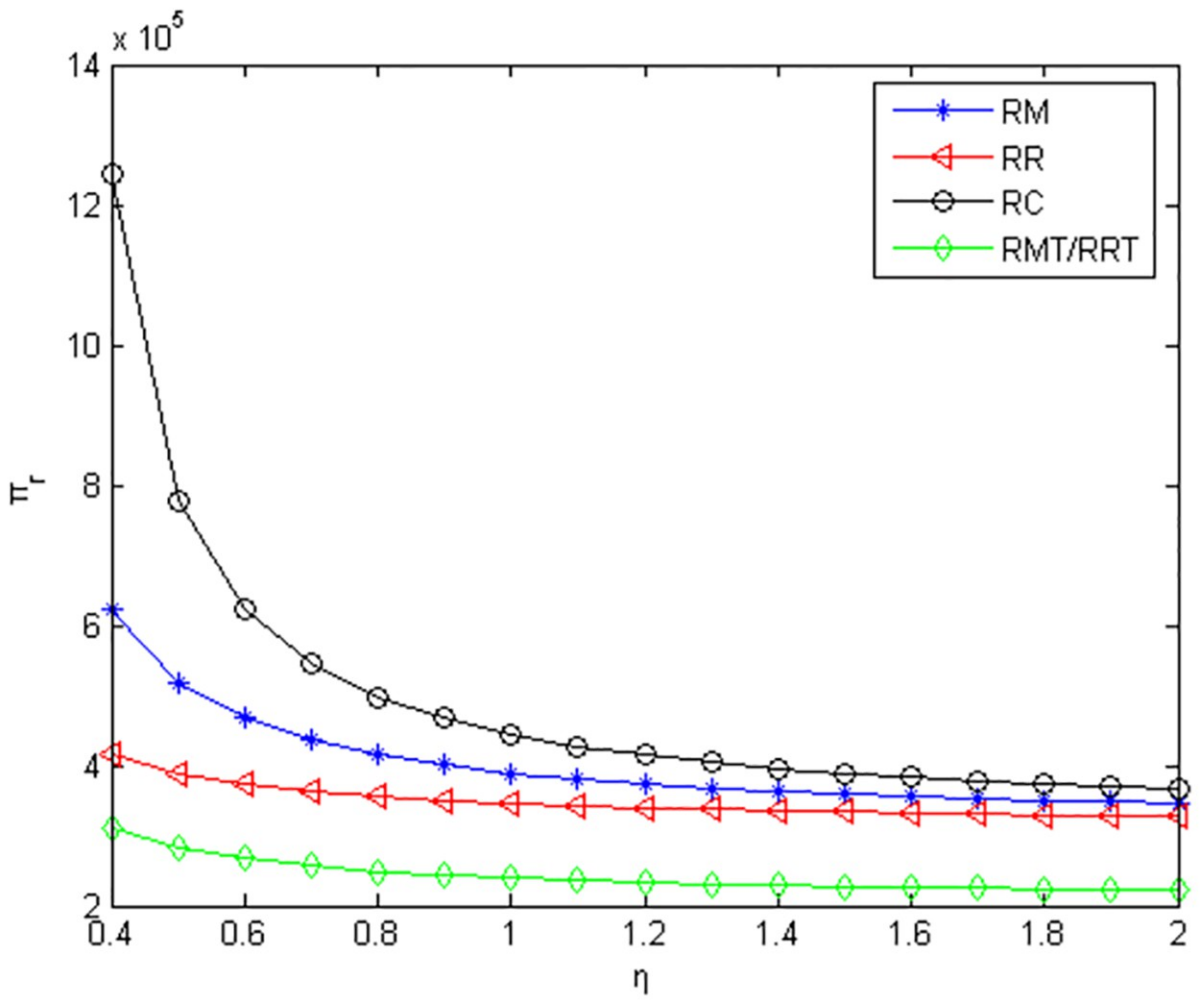
Retailers’ profits in the five R&D modes when retailers are core enterprises.

**Fig 6:**
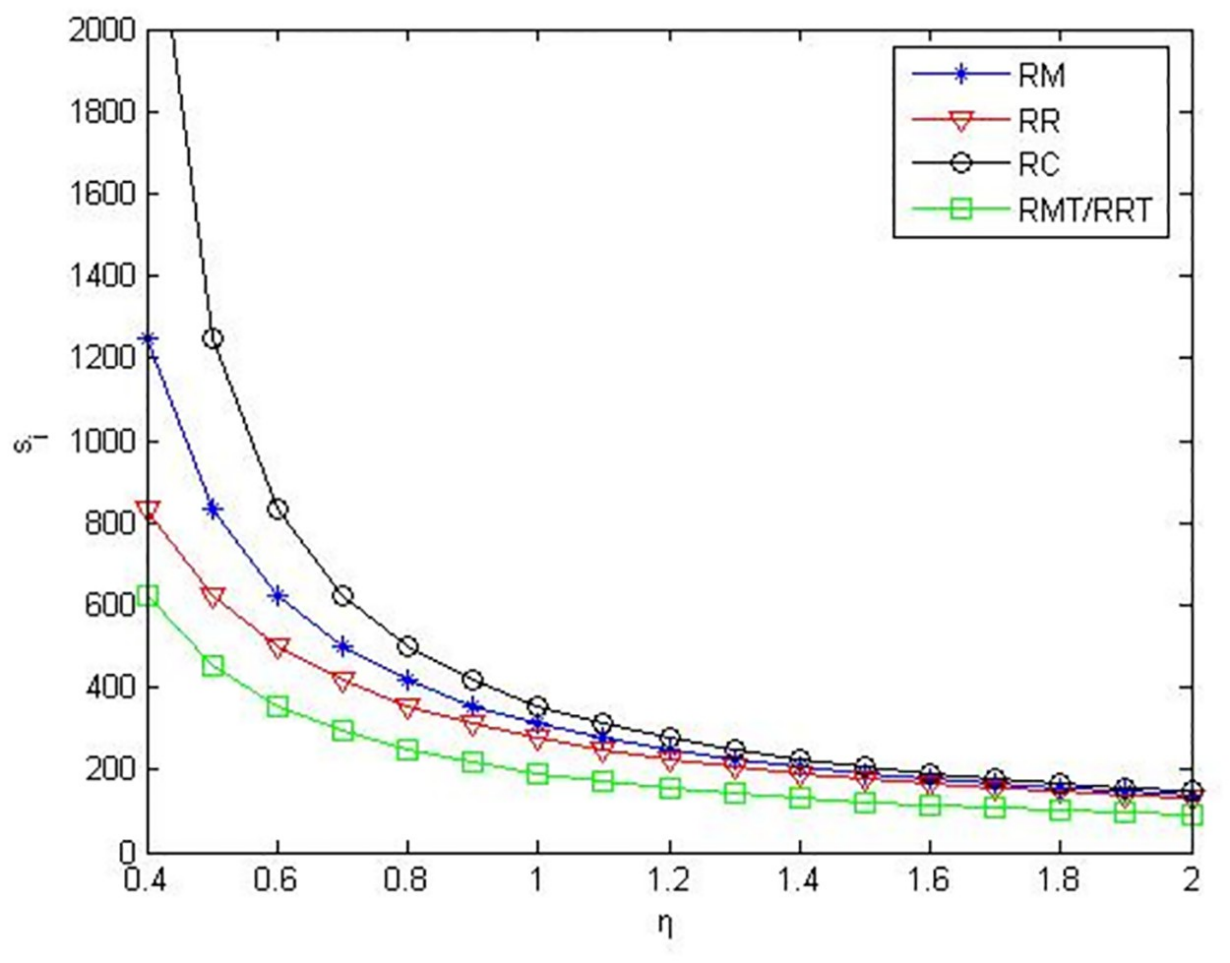
Product green levels in the five R&D modes when retailers are core enterprises.

From Figs 4–6, it can be seen when retailers are core enterprises, compared to retailers’ green product R&D, when manufacturers conduct green product R&D, manufacturers’ profits, retailers’ profits, and product green level are greater. Compared to manufacturers’ or retailers’ green product R&D, when manufacturers and retailers jointly conduct green product R&D, manufacturers’ profits, retailers’ profits, and product green level are higher. Compared to manufacturers outsourcing green product R&D to third-party companies, manufacturers’ profits, retailers’ profits and product green level are greater when manufacturers conduct green product R&D. Compared to retailers outsourcing green product R&D to third-party companies, manufacturers’ profits, retailers’ profits and product green level are greater when retailers conduct green product R&D.

**Fig 7:**
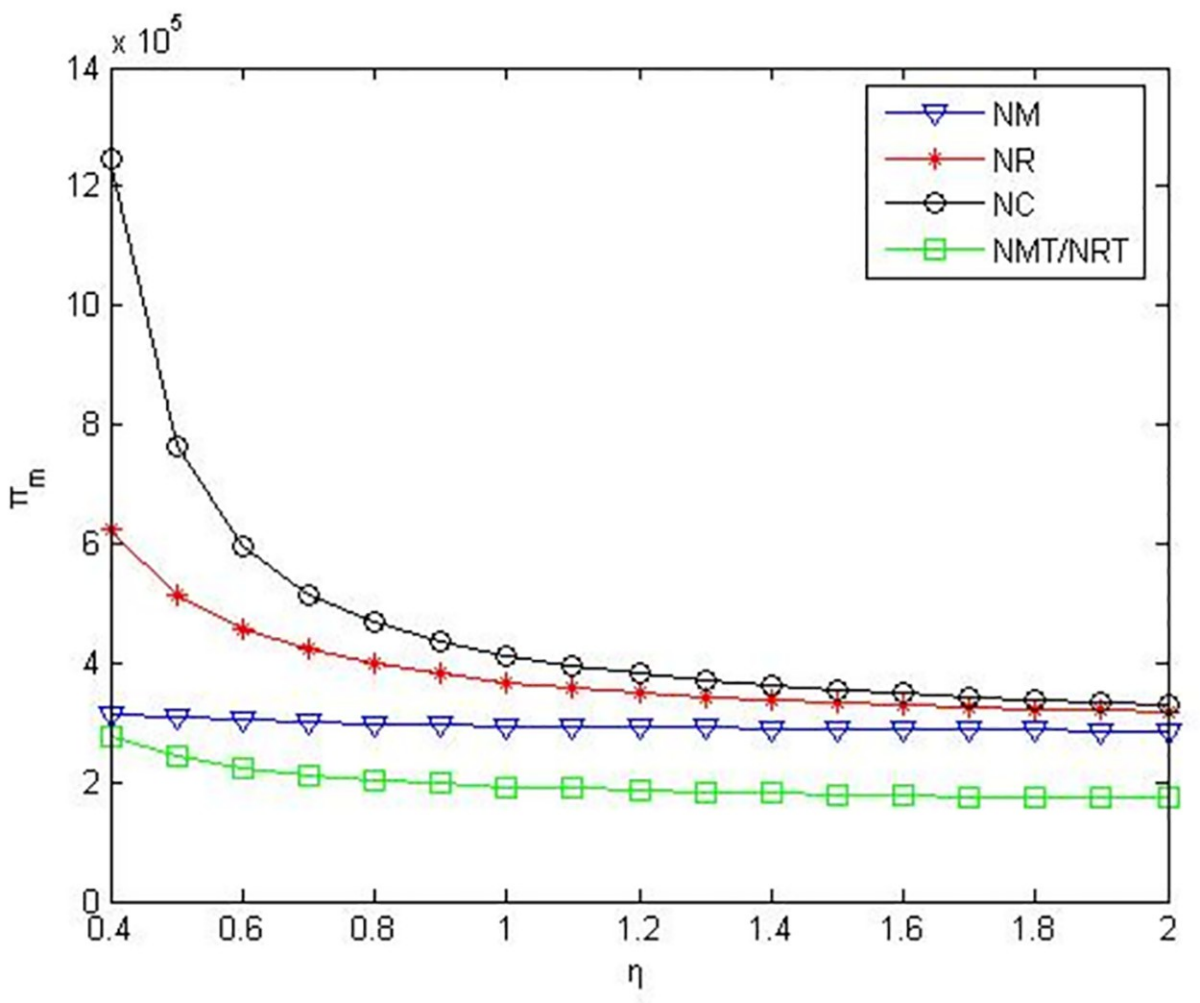
Manufacturers’ profits in the five R&D modes when manufacturers and retailers have equal power.

**Fig 8:**
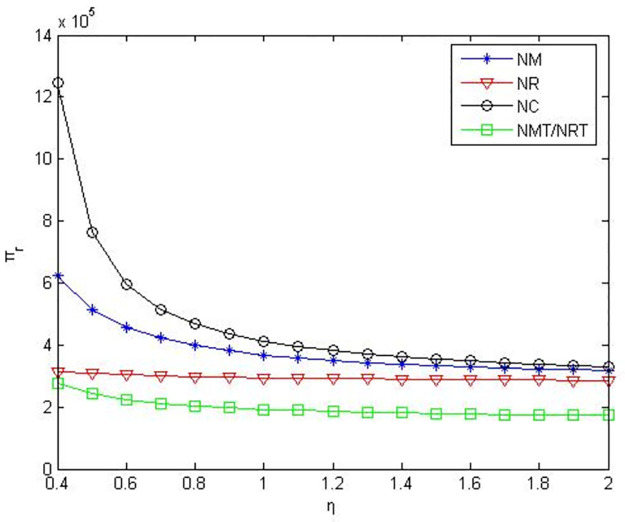
Retailers’ profits in the five R&D modes when manufacturers and retailers have equal power.

**Fig 9:**
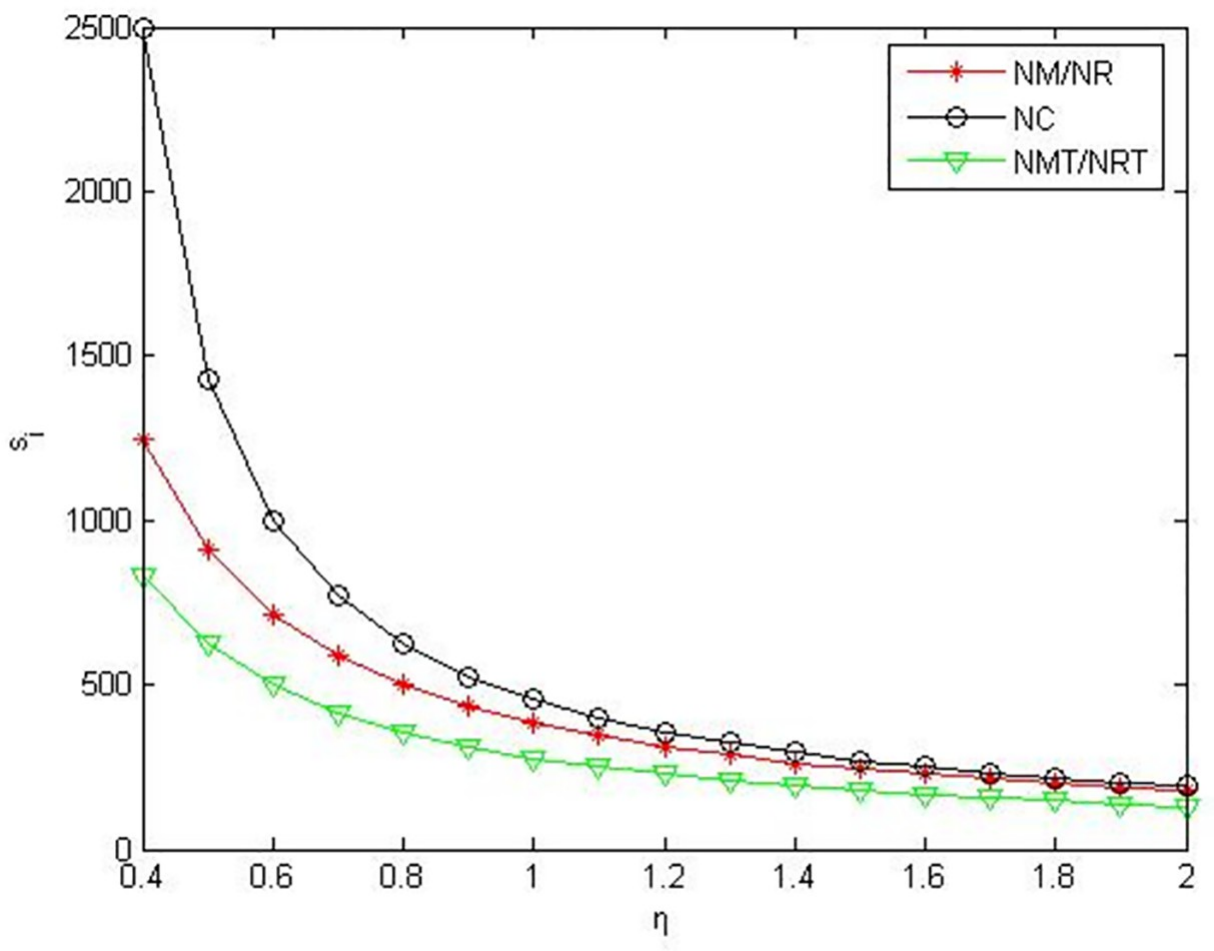
Product green levels in the five R&D modes when manufacturers and retailers have equal power.

It can be seen from Figs 7–9 that when the power of manufacturers and retailers is equal, compared to manufacturers or retailers’ green product R&D, when manufacturers and retailers jointly conduct green product R&D, manufacturers’ profits, retailers’ profits, and product green level are higher. Compared to manufacturers outsourcing green product R&D to third-party companies, manufacturers’ profits, retailers’ profits and product green level are greater when manufacturers conduct green product R&D. Compared to retailers outsourcing green product R&D to third-party companies, manufacturers’ profits, retailers’ profits and product green level are greater when retailers conduct green product R&D.

**Fig 10:**
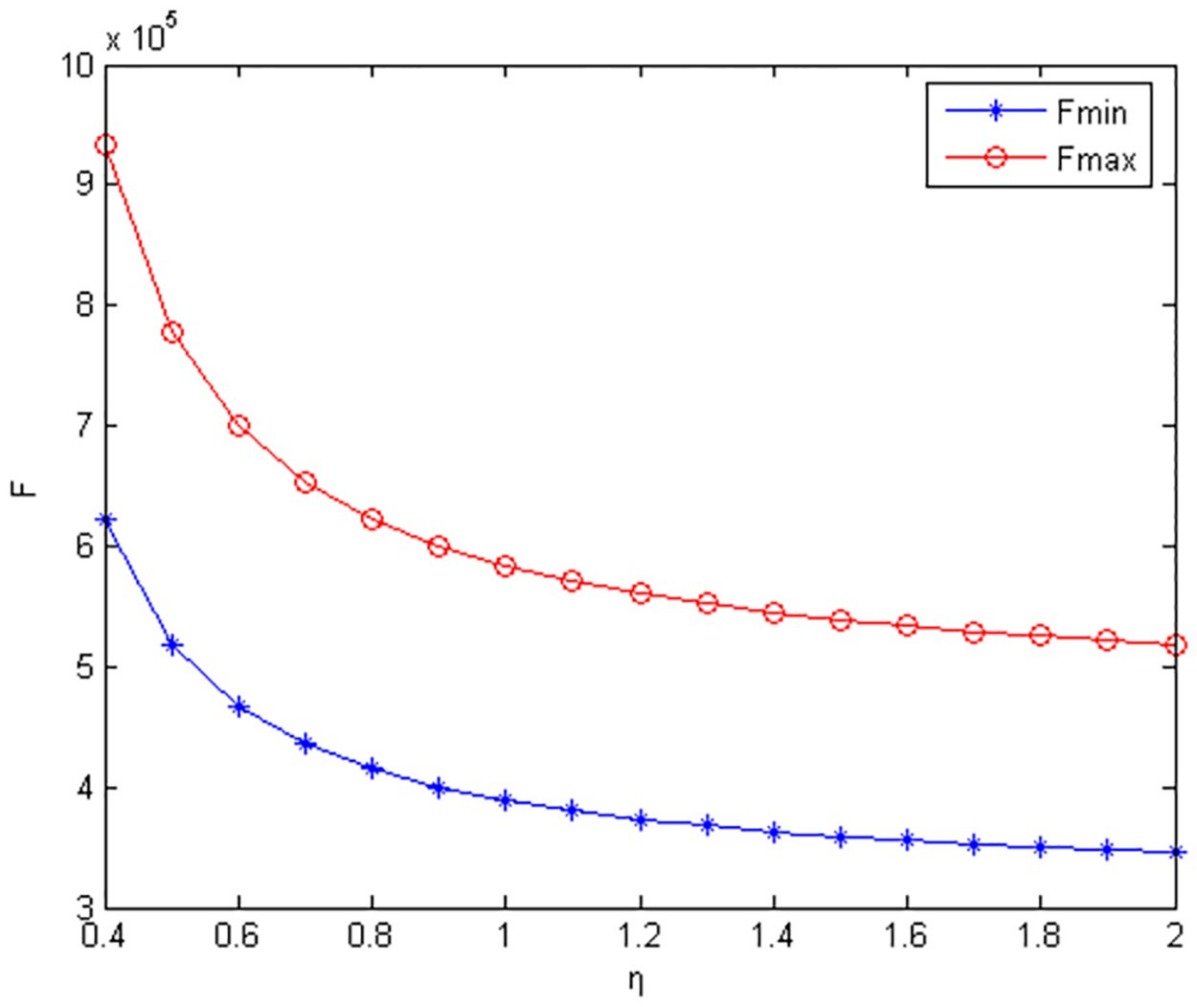
Scope of fixed fees for green product R&D in MR /RM mode.

**Fig 11:**
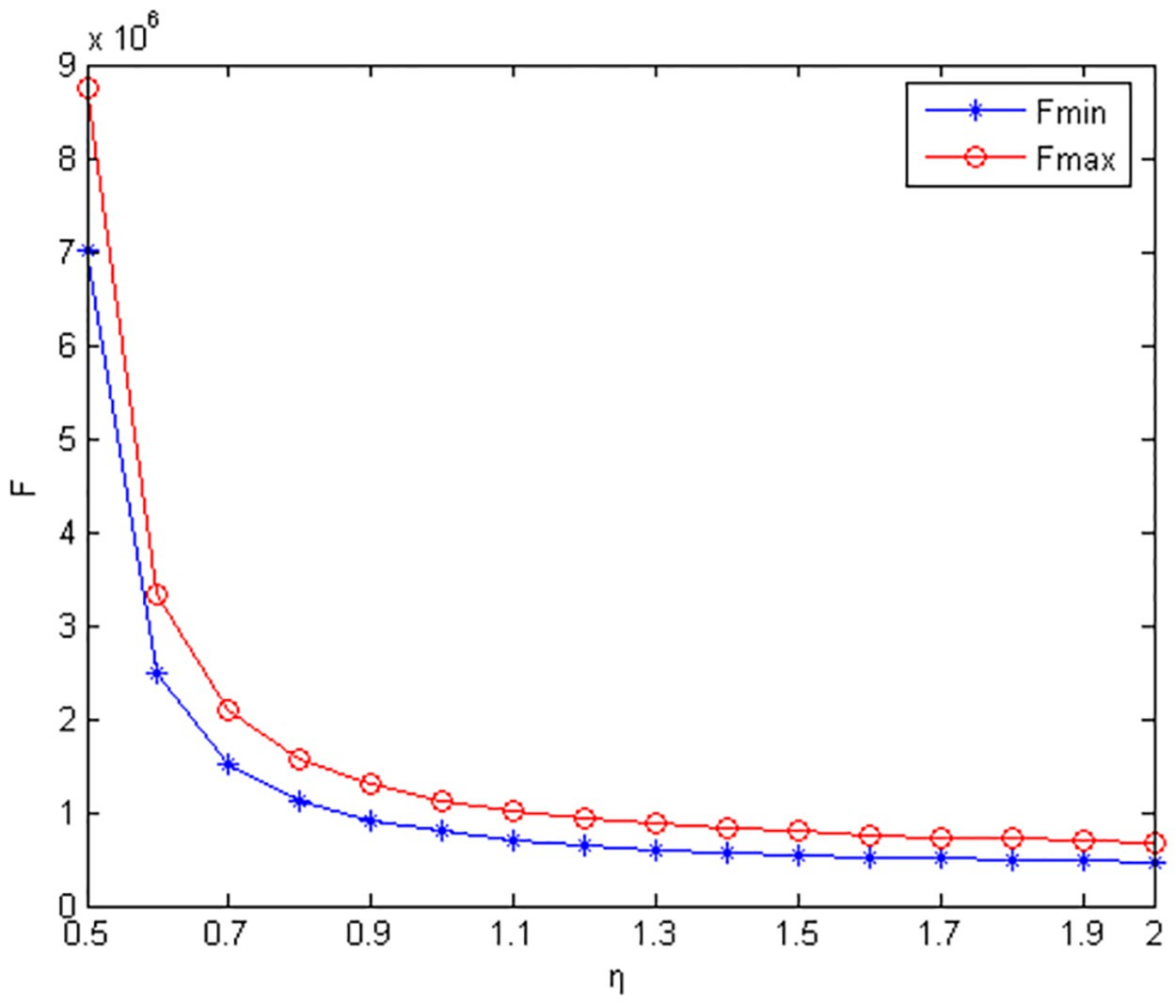
Scope of fixed fees for product green R&D in MC /RC mode.

**Fig 12:**
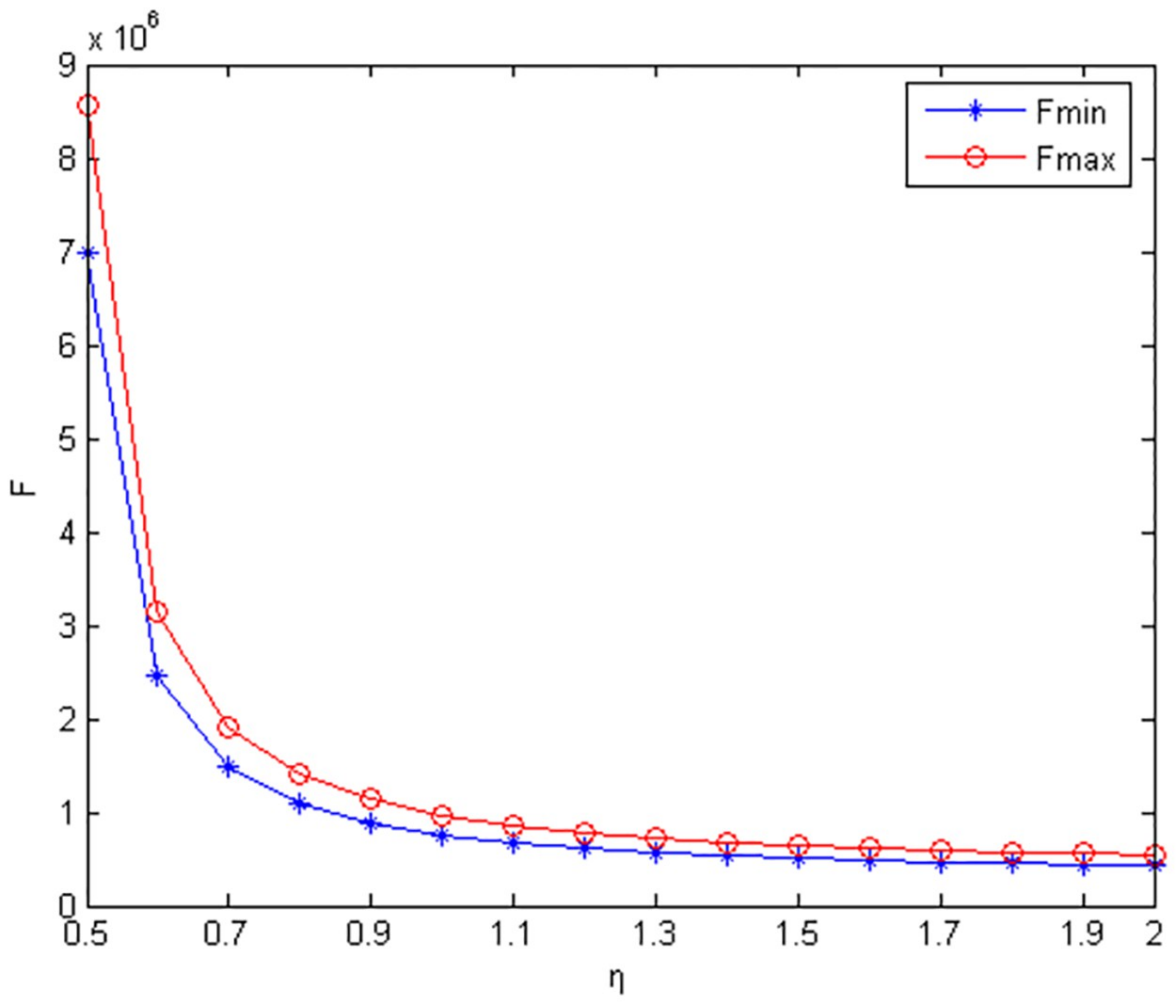
Scope of fixed fees for green product R&D in NC mode.

Figs 10–12 show the scope of fixed fees for two-part pricing contracts coordination in five green product R&D modes.

**Fig 13:**
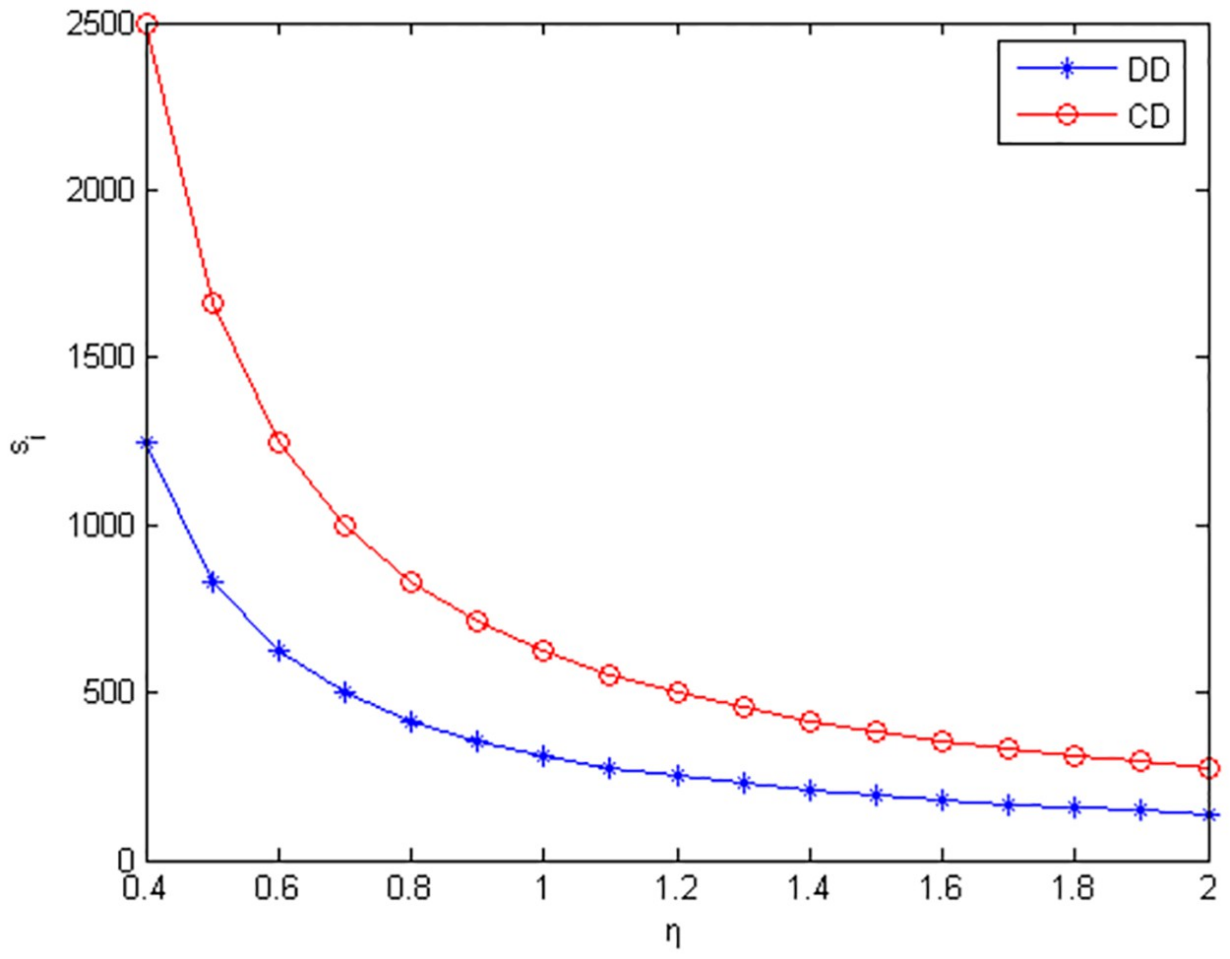
Product green levels of two kinds of decisions in MR /RM mode.

**Fig 14:**
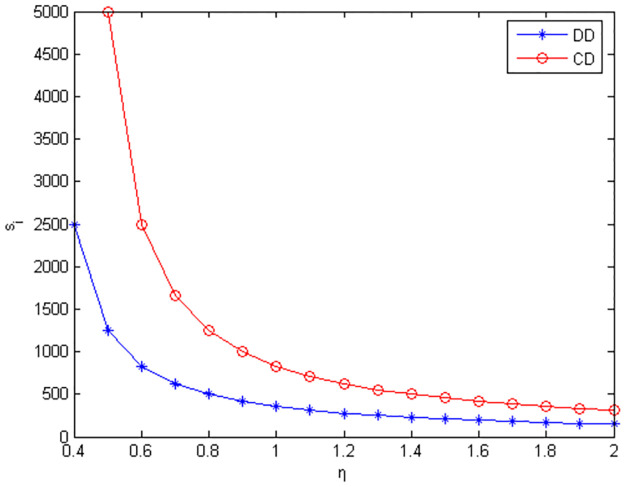
Product green levels of two kinds of decisions in MC /RC mode.

**Fig 15:**
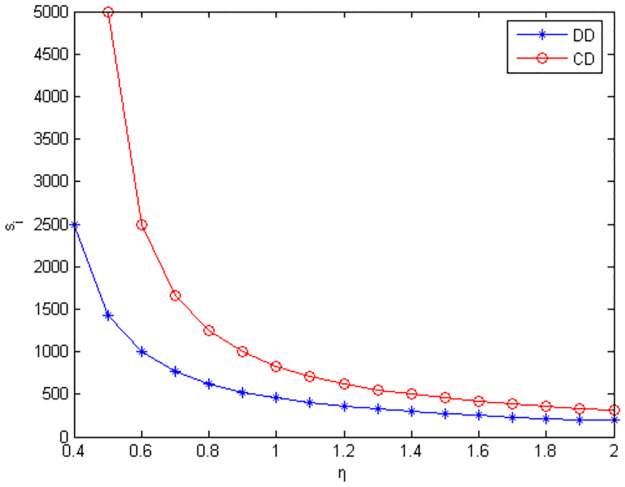
Product green levels in two kinds of decisions in NC mode.

Figs 13–15 show the comparison of the product green level of the five R&D modes in decentralized decision-making and coordinated decision-making of two-part pricing contracts, it can be seen that the product green level of coordinated decision-making is greater than that in decentralized decision-making.

## 8. Conclusion

In the sustainable supply chain, this paper analyzes the optimal mode selection of five green product R&D modes, including green product R&D by manufacturers, green product R&D by retailers, green product R&D by manufacturers outsourcing to third-party companies, green product R&D by retailers outsourcing to third-party companies, and green product R&D by manufacturers and retailers in the three power structures of manufacturers as core enterprises, retailers as core enterprises, and equal power between manufacturers and retailers. It is concluded that the optimal green product R&D mode is that manufacturer and retailer jointly conduct green product R&D. Manufacturers’ green product R&D is better than manufacturers’ outsourcing, and retailers’ green product R&D is better than manufacturers’ outsourcing.

From the perspective of power structures, the equilibrium results of the game model are analyzed, and it is found that power structures have a significant impact on market demand, product green level, product price, enterprise profits and the choice of green product R&D mode. When the power of manufacturers and retailers is equal, the market demand and product green level are the highest and the product price is the lowest. When manufacturers are core enterprises, manufacturers’ profits are the highest, and retailers’ green product R&D is better than the manufacturers’ green product R&D. When retailers are core enterprises, retailers’ profits are the highest, and manufacturers’ green product R&D is better than retailers’ green product R&D. Two-part pricing contracts can achieve supply chain coordination.

### 8.1 Theoretical results

1. If manufacturers or retailers conduct green product R&D, when manufacturers are core enterprises, it is optimal for retailers to conduct green product R&D. When retailers are core enterprises, it is optimal for manufacturers to conduct green product R&D. When manufacturers and retailers have equal power, there is no optimal mode.
2. In the same power structures, manufacturers’ green product R&D is better than manufacturers outsourcing green product R&D to third-party companies. Retailers’ green product R&D is better than outsourcing green product R&D to third-party companies. Manufacturers and retailers jointly conduct green product R&D better than that of manufacturers or retailers.
3. In the same green product R&D mode, when manufacturers and retailers have equal power, the market demand and product green level are the highest, while the product retail price is the lowest. When manufacturers are core enterprises, manufacturers’ profits are the highest. When retailers are core enterprises, retailers’ profits are the highest.
4. For the optimal selection strategy MR (manufacturers are core enterprises, and retailers conduct green product R&D), the supply chain coordination can be achieved by two-part pricing contracts. The coordination strategy is that manufacturers’ wholesale price is equal to the unit cost of products, and retailers pay manufacturers a subsidy of fixed fee F, where $\frac{{\left(D_{0}-\alpha c_{1}\right)}^{2}\eta }{4(2\alpha \eta -\gamma^{2})}\leq F\leq \frac{{{3(D}_{0}-\alpha c_{1})}^{2}\eta }{8\left(2\alpha \eta -\gamma^{2}\right)}$.
5. For the optimal selection strategy RM (retailers are core enterprises, and manufacturers conduct green product R&D), supply chain coordination can be achieved by adopting two-part pricing contracts. The coordination strategy is that the wholesale price of manufacturers is equal to the retail price of the products, and manufacturers pay retailers a subsidy of fixed fee F, where $\frac{{\left(D_{0}-\alpha c_{1}\right)}^{2}\eta }{4(2\alpha \eta -\gamma^{2})}\leq F\leq \frac{{{3(D}_{0}-\alpha c_{1})}^{2}\eta }{8\left(2\alpha \eta -\gamma^{2}\right)}$.
6. For the optimal selection strategy MC (manufacturers are core enterprises, and manufacturers and retailers jointly conduct green product R&D), the supply chain coordination can be achieved by adopting two-part pricing contracts. The coordination strategy is that manufacturers’ wholesale price is equal to the unit cost of products, and retailers pay manufacturers a subsidy of fixed fee F, where $\frac{{\left(D_{0}-\alpha c_{1}\right)}^{2}{(2\alpha \eta -\gamma^{2})\;}^{2}\eta }{8{\left(\alpha \eta -\gamma^{2}\right)}^{2}(4\alpha \eta -3\gamma^{2})\;}\leq F\leq \frac{{{(D}_{0}-\alpha c_{1})}^{2}{(2\alpha \eta -\gamma^{2})\;}^{2}(6\alpha \eta -5\gamma^{2})\;\eta }{8{\left(\alpha \eta -\gamma^{2}\right)}^{2}{(4\alpha \eta -3\gamma^{2})\;}^{2}}$
7. For the optimal selection strategy RC (retailers are core enterprises, manufacturers and retailers jointly conduct green product R&D), supply chain coordination can be achieved by using two-part pricing contracts. The coordination strategy is that the wholesale price of manufacturers is equal to the retail price of products, and manufacturers pay retailers a subsidy of fixed fee F, where $\frac{{\left(D_{0}-\alpha c_{1}\right)}^{2}{(2\alpha \eta -\gamma^{2})\;}^{2}\eta }{8{\left(\alpha \eta -\gamma^{2}\right)}^{2}(4\alpha \eta -3\gamma^{2})\;}\leq F\leq \frac{{{(D}_{0}-\alpha c_{1})}^{2}{(2\alpha \eta -\gamma^{2})\;}^{2}(6\alpha \eta -5\gamma^{2})\;\eta }{8{\left(\alpha \eta -\gamma^{2}\right)}^{2}{(4\alpha \eta -3\gamma^{2})\;}^{2}}$.
8. For the optimal selection strategy NC (equal power between manufacturers and retailers, and manufacturers and retailers jointly conduct green product R&D), when certain conditions are met, the supply chain coordination can be achieved by using two-pricing contracts. The coordination strategy is that the wholesale price of manufacturers is equal to the retail price of products, and manufacturers pay retailers a subsidy of fixed fee F, where $\frac{{\left(D_{0}-\alpha c_{1}\right)}^{2}(4\gamma^{4}-11\gamma^{2}\alpha \eta +8\alpha^{2}\eta^{2})\alpha \eta^{2}}{8{\left(\alpha \eta -\gamma^{2}\right)}^{2}{\left(3\alpha \eta -2\gamma^{2}\right)}^{2}}\leq F\leq \frac{{{(D}_{0}-\alpha c_{1})}^{2}(2\alpha \eta -\gamma^{2})(5\alpha \eta -4\gamma^{2})\alpha \eta^{2}}{8{\left(\alpha \eta -\gamma^{2}\right)}^{2}{\left(3\alpha \eta -2\gamma^{2}\right)}^{2}}$.

### 8.2 Management implications

Based on the above conclusions, the following important management implications can guide enterprises to make more reasonable decisions in the choice of green product R&D mode of sustainable supply chain.

1. In different power structures, when manufacturers are core enterprises, retailers’ green product R&D is superior to that of manufacturers. When retailers are core enterprises, manufacturers’ green product R&D is superior to that of retailers. In the same power structure, manufacturers’ green product R&D is better than manufacturers outsourcing green product R&D to third-party companies. Retailers’ green product R&D is better than outsourcing green product R&D to third-party companies. Manufacturers and retailers jointly conduct green product R&D better than that of manufacturers or retailers.
2. Green product R&D is crucial for the sustainable development of enterprises. Different enterprises conduct green product R&D from different aspects, such as production process, sales, packaging, which may affect the product price, green level and profits of the supply chain. While green product R&D enables enterprises to protect the environment and improve product competitiveness. It also requires enterprises to invest more costs and take more risks. To promote the progress of green product R&D, government can provide subsidies or enterprises adopt the cost-sharing measures.
3. The power structure of the supply chain has a significant impact on supply chain decision-making. Previous studies have shown that dominant enterprises have a dominant advantage and can obtain greater profits. Among the five green product R&D modes, core enterprises also have a leading advantage. When manufacturers are core enterprises, manufacturers’ profits are the highest, and when retailers are core enterprises, retailers’ profits are the highest.
4. In the five optimal selection strategies of green product R&D, the two-part pricing contracts can realize supply chain coordination. Of course, managers can also explore the coordination of joint contracts such as profit-sharing and cost-sharing contracts.

### 8.3 Limitation and future research

By establishing a game model, this paper obtains the optimal green product R&D mode of sustainable supply chains in different power structures, but there are still some limitations: firstly, it needs to pay more cost and bear greater risks to conduct green product R&D, then the choice of green product R&D modes for cost-sharing between manufacturers and retailers or government subsidies can be considered in the future. Secondly, green products R&D will be affected by market competition, so it is necessary to consider the competition of price and green level in the future. Finally, this paper applies two-part pricing contracts to supply chain coordination, and other contracts such as revenue-sharing and cost-sharing can also be considered.

## Supporting information

S1 Data (DOCX)

S1 Appendix (DOCX)
