# Supplementary material for: R&D mode and coordination of green products in sustainable supply chain considering power structures
Source: PLoS One. 2023 Nov 2;18(11):e0291351. doi: 10.1371/journal.pone.0291351 (PMC10621872; doi:10.1371/journal.pone.0291351)
Supplement: S1 Data — (DOCX) [file pone.0291351.s001.docx]

**Data set**

We establish game models to study the choice of green product R&D modes in different power structures and the influence of power structure on the optimal decision of sustainable supply chain. In the numerical analysis of this paper, the original data of parameters are$\alpha=0.1, c_{1}=10, D_{0}=500, \gamma=0.2$.
