# Supplementary material for: R&D mode and coordination of green products in sustainable supply chain considering power structures
Source: PLoS One. 2023 Nov 2;18(11):e0291351. doi: 10.1371/journal.pone.0291351 (PMC10621872; doi:10.1371/journal.pone.0291351)
Supplement: S1 Appendix — (DOCX) [file pone.0291351.s002.docx]

Appendix

**Proof of Theorem 1.**

When$2\alpha\eta-\gamma^{2}>0$, from $D^{\mathrm{MR}}\geq D^{\mathrm{MM}},$we can obtain $\frac{{(D}_{0}-\alpha c_{1})\alpha\eta}{4\alpha\eta-2\gamma^{2}}\geq\frac{{(D}_{0}-\alpha c_{1})\alpha\eta}{4\alpha\eta-\gamma^{2}}$. From$s_{r}^{\mathrm{MR}}\geq s_{m}^{\mathrm{MM}}$, $\frac{\left( D_{0}-\alpha c_{1} \right)\gamma}{\text{4}\alpha\eta-2\gamma^{2}}\geq\frac{\left( {(D}_{0}-\alpha c_{1} \right)\gamma}{\text{4}\alpha\eta-\gamma^{2}}$ is true. From $\pi_{m}^{\mathrm{MR}}\geq\pi_{m}^{\mathrm{MM}}$, $\frac{{{(D}_{0}-\alpha c_{1})}^{2}\eta}{\text{4(2}\alpha\eta-\gamma^{2})}\geq\frac{{{(D}_{0}-\alpha c_{1})}^{2}\eta}{\text{2(4}\alpha\eta-\gamma^{2})}$ is true. From $\pi_{r}^{\mathrm{MR}}\geq\pi_{r}^{\mathrm{MM}}, \frac{{{(D}_{0}-\alpha c_{1})}^{2}\eta}{8\left( 2\alpha\eta-\gamma^{2} \right)}\geq\frac{{{(D}_{0}-\alpha c_{1})}^{2}\alpha\eta^{2}}{\left( 4\alpha\eta-\gamma^{2} \right)^{2}}$, simplified to $r^{4}\geq0$，then the inequalityis true. From $\text{p}_{\text{1}}^{\text{MR}}\leq\text{p}_{\text{1}}^{\text{MM}}, \frac{\text{(3}\alpha\eta-\gamma^{2}\text{)}{(D}_{0}\text{+(}\alpha\eta-\gamma^{2}\text{)}\alpha c_{1}}{\text{2}\alpha\text{(2}\alpha\eta-\gamma^{2})}\leq\frac{3D_{0}\eta+\left( \alpha\eta-\gamma^{2} \right)c_{1}}{4\alpha\eta-\gamma^{2}}$, simplified to $\text{(}\alpha\eta-\gamma^{2}\text{)}{(D}_{0}-\alpha c_{1}) \geq0,$then when $\alpha\eta-\gamma^{2}\geq0$, the inequality is true.

Then, Theorem 1 is proven.

**Proof of Theorem 2.**

When$4\alpha\eta{-3\gamma}^{2}>0$, from $D^{\mathrm{MC}}\geq D^{\mathrm{MR}},$we can obtain$\frac{{(D}_{0}-\alpha c_{1})\alpha\eta}{4\alpha\eta-3\gamma^{2}}\geq\frac{{(D}_{0}-\alpha c_{1})\alpha\eta}{4\alpha\eta-2\gamma^{2}}$. From${s_{r}^{\mathrm{MC}}\geq s}_{r}^{\mathrm{MR}}$, $\frac{\left( {(D}_{0}-\alpha c_{1} \right)\gamma}{\text{4}\alpha\eta-3\gamma^{2}}\geq\frac{\left( {(D}_{0}-\alpha c_{1} \right)\gamma}{\text{4}\alpha\eta-2\gamma^{2}}$is true. From$\pi_{m}^{\mathrm{MC}}\geq\pi_{m}^{\mathrm{MR}}$, $\frac{{{(D}_{0}-\alpha c_{1})}^{2}\eta}{\text{2(4}\alpha\eta-3\gamma^{2})}\geq\frac{{{(D}_{0}-\alpha c_{1})}^{2}\eta}{\text{4(2}\alpha\eta-\gamma^{2})}$is true. From$\pi_{r}^{\mathrm{MC}}{\geq\pi}_{r}^{\mathrm{MR}}$ $, \frac{{{(D}_{0}-\alpha c_{1})}^{2}\left( 2\alpha\eta-\gamma^{2} \right)\eta}{\text{2}{\text{(4}\alpha\eta-3\gamma^{2})}^{2}}\geq\frac{{{(D}_{0}-\alpha c_{1})}^{2}\eta}{8\left( 2\alpha\eta-\gamma^{2} \right)}$, simplified to $8\alpha\eta-5\gamma^{2}\geq0$，then the inequalityis true. From $\text{p}_{\text{1}}^{\text{MC}}\leq\text{p}_{\text{1}}^{\text{MR}}, \frac{{(D}_{0}(\text{3}\alpha\eta\text{-}\gamma^{2}\text{)}+(\alpha\eta-2\gamma^{2})\alpha c_{1}}{\text{α(4}\alpha\eta-3\gamma^{2})}\leq\frac{\text{(3}\alpha\eta-\gamma^{2}\text{)}{(D}_{0}\text{+(}\alpha\eta-\gamma^{2}\text{)}\alpha c_{1}}{\text{2α(2}\alpha\eta-\gamma^{2})}$, simplified to $r^{2}\geq0,$then the inequality is true.

Then, Theorem 2 is proven.

**Proof of Theorem 3.**

When$2\alpha\eta{-\gamma}^{2}\geq0$, from $D^{\mathrm{MM}}\geq D^{\mathrm{MMT}},$we can obtain $\frac{{(D}_{0}-\alpha c_{1})\alpha\eta}{4\alpha\eta-\gamma^{2}}\geq\frac{{(D}_{0}-\alpha c_{1})\alpha\eta}{2(3\alpha\eta-\gamma^{2})}$. From$s_{m}^{\mathrm{MM}}\geq s_{t}^{\mathrm{MMT}}$, $\frac{{(D}_{0}-\alpha c_{1})\gamma}{4\alpha\eta-\gamma^{2}}\geq\frac{{(D}_{0}-\alpha c_{1})\gamma}{2(3\alpha\eta-\gamma^{2})}$is true. From$\pi_{m}^{\mathrm{MM}}\geq\pi_{m}^{\mathrm{MMT}}$, ，$\frac{{{(D}_{0}-\alpha c_{1})}^{2}\eta}{2\left( 4\alpha\eta-\gamma^{2} \right)}\geq\frac{{{(D}_{0}-\alpha c_{1})}^{2}\eta}{4(3\alpha\eta-\gamma^{2})}$is true. From$\pi_{r}^{\mathrm{MM}}\geq\pi_{r}^{\mathrm{MMT}}$ $, \frac{{{(D}_{0}-\alpha c_{1})}^{2}\alpha\eta^{2}}{\left( 4\alpha\eta-\gamma^{2} \right)^{2}}\geq\frac{{{(D}_{0}-\alpha c_{1})}^{2}\alpha\eta^{2}}{4{(3\alpha\eta-\gamma^{2})}^{2}}$is true. From $\text{p}_{\text{1}}^{\text{MM}}\leq\text{p}_{\text{1}}^{\text{MMT}}, \frac{3D_{0}\eta+\left( \alpha\eta-\gamma^{2} \right)c_{1}}{4\alpha\eta-\gamma^{2}}\leq\frac{\left( 5\alpha\eta-\gamma^{2} \right)D_{0}+\left( \alpha\eta-\gamma^{2} \right)\alpha c_{1}}{2\alpha(3\alpha\eta-\gamma^{2})}$, simplified to simplified to $(2\alpha\eta-\gamma^{2}) (\alpha\eta-\gamma^{2}){(D}_{0}\geq$(2$\alpha^{2}\eta^{2}-3\alpha\eta\gamma^{2}$)$\alpha c_{1}$，because $\left( 2\alpha\eta-\gamma^{2} \right)\left( \alpha\eta-\gamma^{2} \right){(D}_{0}\geq\left( 2\alpha\eta-\gamma^{2} \right)\left( \alpha\eta-\gamma^{2} \right)\alpha c_{1}=$(2$\alpha^{2}\eta^{2}-3\alpha\eta\gamma^{2}+\gamma^{4}$)$\alpha c_{1}\geq(2\alpha^{2}\eta^{2}-3\alpha\eta\gamma^{2}) \alpha c_{1},$then when $\alpha\eta-\gamma^{2}\geq0$,the inequality is true.

Then, Theorem 3 is proven.

**Proof of Theorem 4.**

When$2\alpha\eta{-\gamma}^{2}>0$, from $D^{\mathrm{MR}}\geq D^{\mathrm{MRT}},$we can obtain $\frac{{(D}_{0}-\alpha c_{1})\alpha\eta}{\text{2(2}\alpha\eta-\gamma^{2})}\geq\frac{{(D}_{0}-\alpha c_{1})\alpha\eta}{2\left( 3\alpha\eta-\gamma^{2} \right)}$. From$s_{m}^{\mathrm{MR}}\geq s_{t}^{\mathrm{MRT}}$, $\frac{{(D}_{0}-\alpha c_{1}\text{)γ}}{\text{2(2}\alpha\eta-\gamma^{2})}\geq\frac{{(D}_{0}-\alpha c_{1})\gamma}{2\left( 3\alpha\eta-\gamma^{2} \right)}$is true. From$\pi_{m}^{\mathrm{MR}}\geq\pi_{m}^{\mathrm{MRT}}$, $\frac{\left( D_{0}-\alpha c_{1} \right)^{2}\eta}{4\left( 2\alpha\eta-\gamma^{2} \right)}\geq\frac{{{(D}_{0}-\alpha c_{1})}^{2}\eta}{4\left( 3\alpha\eta-\gamma^{2} \right)}$is true. From$\pi_{r}^{\mathrm{MR}}\geq\pi_{r}^{\mathrm{MRT}}$ $, \frac{{{(D}_{0}-\alpha c_{1})}^{2}\eta}{8\left( 2\alpha\eta-\gamma^{2} \right)}\geq\frac{{{(D}_{0}-\alpha c_{1})}^{2}\alpha\eta^{2}}{4\left( 3\alpha\eta-\gamma^{2} \right)^{2}} ,$ simplified to ${(2\alpha\eta-\gamma^{2})}^{2}+\alpha^{2}\eta^{2}\geq$0 is true. From $\text{p}_{\text{1}}^{\text{MR}}\leq\text{p}_{\text{1}}^{\text{MRT}}, \frac{\text{(3}\alpha\eta-\gamma^{2}\text{)}{(D}_{0}\text{+(}\alpha\eta-\gamma^{2}\text{)}\alpha c_{1}}{\text{2α(2}\alpha\eta-\gamma^{2})}\leq\frac{\left( 5\alpha\eta-\gamma^{2} \right)D_{0}+\left( \alpha\eta-\gamma^{2} \right)\alpha c_{1}}{2\alpha(3\alpha\eta-\gamma^{2})}$, simplified to $(\alpha\eta-\gamma^{2}){(D}_{0}\geq$($\alpha\eta-\gamma^{2}$)$\alpha c_{1}$,then when $\alpha\eta-\gamma^{2}\geq0$,the inequality is true.

Then, Theorem 4 is proven.

**Proof of Theorem 5.**

Because$D^{\mathrm{RM}}=D^{\mathrm{MR}}$，$D^{\mathrm{RR}}=D^{\mathrm{MM}}$，$s_{r}^{\mathrm{RM}}=s_{r}^{\mathrm{MR}}，{s_{m}^{\mathrm{RR}}=s}_{m}^{\mathrm{MM}}$，${\pi_{m}^{\mathrm{RM}}=\pi}_{m}^{\mathrm{MR}}，\pi_{m}^{\mathrm{RR}}=\pi_{m}^{\mathrm{MM}}$，${\pi_{r}^{\mathrm{RM}}=\pi}_{r}^{\mathrm{MR}}，\pi_{r}^{\mathrm{RR}}=\pi_{r}^{\mathrm{MM}}$，$\text{p}_{\text{1}}^{\text{RR}}=\text{p}_{\text{1}}^{\text{MM}}，\text{p}_{\text{1}}^{\text{MR}}=\text{p}_{\text{1}}^{\text{RM}}$, it can be proved in the same way as Theorem 1.

Then, Theorem 5 is proven.

**Proof of Theorem 6.**

Because$D^{\mathrm{RC}}=D^{\mathrm{MC}}$，$D^{\mathrm{RM}}=D^{\mathrm{MR}}$，$s_{r}^{\mathrm{RC}}=s_{r}^{\mathrm{MC}}{，s}_{r}^{\mathrm{RM}}=s_{r}^{\mathrm{MR}}$，$\pi_{m}^{\mathrm{RC}}=\pi_{m}^{\mathrm{MC}}$，${\pi_{m}^{\mathrm{RM}}=\pi}_{m}^{\mathrm{MR}}$，$\pi_{r}^{\mathrm{RC}}=\pi_{r}^{\mathrm{MC}}$，${\pi_{r}^{\mathrm{RM}}=\pi}_{r}^{\mathrm{MR}}，\text{p}_{\text{1}}^{\text{RC}}=\text{p}_{\text{1}}^{\text{MC}}，\text{p}_{\text{1}}^{\text{MR}}=\text{p}_{\text{1}}^{\text{RM}}$, it can be proved in the same way as Theorem 2.

Then, Theorem 6 is proven.

**Proof of Theorem 7.**

Because$D^{\mathrm{RM}}=D^{\mathrm{MR}}$，$s_{r}^{\mathrm{RM}}=s_{r}^{\mathrm{MR}}$，${\pi_{m}^{\mathrm{RM}}=\pi}_{m}^{\mathrm{MR}}$，${\pi_{r}^{\mathrm{RM}}=\pi}_{r}^{\mathrm{MR}}$，$\text{p}_{\text{1}}^{\text{MR}}=\text{p}_{\text{1}}^{\text{RM}}，D^{\mathrm{RMT}}=D^{\mathrm{MRT}}$，$s_{t}^{\mathrm{RMT}}=s_{t}^{\mathrm{MRT}}$，$\pi_{m}^{\mathrm{RMT}}=\pi_{m}^{\mathrm{MRT}}$，$\pi_{r}^{\mathrm{RMT}}=\pi_{r}^{\mathrm{MRT}}$, it can be proved in the same way as Theorem 4.

Then, Theorem 7 is proven.

**Proof of Theorem 8.**

Because $D^{\mathrm{RR}}=D^{\mathrm{MM}}$，$s_{r}^{\mathrm{RR}}=s_{r}^{\mathrm{MM}}$，${\pi_{m}^{\mathrm{RR}}=\pi}_{m}^{\mathrm{MM}}$，${\pi_{r}^{\mathrm{RR}}=\pi}_{r}^{\mathrm{MM}}$，$\text{p}_{\text{1}}^{\text{RR}}=\text{p}_{\text{1}}^{\text{RM}}$,$D^{\mathrm{RRT}}=D^{\mathrm{MMT}}$，$s_{t}^{\mathrm{RRT}}=s_{t}^{\mathrm{MMT}}$，$\pi_{m}^{\mathrm{RRT}}=\pi_{m}^{\mathrm{MMT}}$，$\pi_{r}^{\mathrm{RRT}}=\pi_{r}^{\mathrm{MMT}}$, it can be proved in the same way as Theorem 3.

Then, Theorem 8 is proven.

**Proof of Theorem 9.**

When$3\alpha\eta-2\gamma^{2}>0$, from $D^{\mathrm{NC}}\geq D^{\mathrm{NR}}=D^{\mathrm{NM}},$we can obtain $\frac{{(D}_{0}-\alpha c_{1})\alpha\eta}{3\alpha\eta-2\gamma^{2}}\geq\frac{{(D}_{0}-\alpha c_{1})\alpha\eta}{3\alpha\eta-\gamma^{2}}$. From$s_{r}^{\mathrm{NC}}\geq s_{r}^{\mathrm{NR}}=s_{m}^{\mathrm{NM}}$, $\frac{\left( {(D}_{0}-\alpha c_{1} \right)\gamma}{3\alpha\eta-2\gamma^{2}}>\frac{(D_{0}-\alpha c_{1})\gamma}{3\alpha\eta-\gamma^{2}}$is true. From$\pi_{m}^{\mathrm{NR}}\geq\pi_{m}^{\mathrm{NM}}$, $\frac{{{(D}_{0}-\alpha c_{1})}^{2}\alpha\eta^{2}}{\left( 3\alpha\eta-\gamma^{2} \right)^{2}}\geq\frac{{{(D}_{0}-\alpha c_{1})}^{2}\left( 2\alpha\eta-\gamma^{2} \right)\eta}{2\left( 3\alpha\eta-\gamma^{2} \right)^{2}},$simplified to $\gamma^{2}\geq0$ is true. From$\pi_{m}^{\mathrm{NC}}{\geq\pi}_{m}^{\mathrm{NR}}$ $, \frac{{{(D}_{0}-\alpha c_{1})}^{2}(2\alpha\eta-\gamma^{2})\eta}{2\left( 3\alpha\eta-2\gamma^{2} \right)^{2}}\geq\frac{{{(D}_{0}-\alpha c_{1})}^{2}\alpha\eta^{2}}{\left( 3\alpha\eta-\gamma^{2} \right)^{2}} ,$ simplified to $3\alpha^{2}\eta^{2}-\gamma^{4}\geq0$is true. From $\text{p}_{\text{1}}^{\text{NC}}\geq\text{p}_{\text{1}}^{\text{NM}}\text{=p}_{\text{1}}^{\text{NR}},\frac{{(D}_{0}\eta+(\alpha\eta-2\gamma^{2})c_{1}}{\text{3}\alpha\eta-2\gamma^{2}}\geq\frac{{2D}_{0}\eta+\left( \alpha\eta-\gamma^{2} \right)c_{1}}{3\alpha\eta-\gamma^{2}}$ is true. Because $\pi_{r}^{\mathrm{NC}}=\pi_{m}^{\mathrm{NC}}$，$\pi_{r}^{\mathrm{NM}}=\pi_{m}^{\mathrm{NR}}$，$\pi_{r}^{\mathrm{NR}}=\pi_{m}^{\mathrm{NM}}$，${\pi_{r}^{\mathrm{NC}}\geq\pi}_{r}^{\mathrm{NM}}{\geq\pi}_{r}^{\mathrm{NR}}$ is true.

Then, Theorem 9 is proven.

**Proof of Theorem 10.**

When$3\alpha\eta-\gamma^{2}>0$, from $D^{\mathrm{NM}}\geq D^{\mathrm{NMT}},$we can obtain $\frac{{(D}_{0}-\alpha c_{1})\alpha\eta}{3\alpha\eta-\gamma^{2}}\geq\frac{{(D}_{0}-\alpha c_{1})\alpha\eta}{4\alpha\eta-\gamma^{2}}$. From$s_{m}^{\mathrm{NM}}\geq s_{t}^{\mathrm{NMT}}$,$\frac{{(D}_{0}-\alpha c_{1})\gamma}{3\alpha\eta-\gamma^{2}}\geq\frac{{(D}_{0}-\alpha c_{1})\gamma}{4\alpha\eta-\gamma^{2}}$is true. From$\pi_{m}^{\mathrm{NM}}\geq\pi_{m}^{\mathrm{NMT}}$, $\frac{{{(D}_{0}-\alpha c_{1})}^{2}(2\alpha\eta-\gamma^{2})\eta}{2\left( 3\alpha\eta-\gamma^{2} \right)^{2}}\leq\frac{{{(D}_{0}-\alpha c_{1})}^{2}\alpha\eta^{2}}{{(4\alpha\eta-\gamma^{2})}^{2}},$simplified to $14\alpha^{3}\eta^{3}-20\alpha^{2}\eta^{2}\gamma^{2}+8\alpha\eta\gamma^{4}+\gamma^{6}=2\alpha\eta\left( 7\alpha\eta-3\gamma^{2} \right)\left( \alpha\eta-\gamma^{2} \right)+\gamma^{4}(2\alpha\eta-\gamma^{2})\geq$0 ,when$\alpha\eta-\gamma^{2}\geq0,$ the inequality is true. From$\pi_{r}^{\mathrm{NM}}\geq\pi_{r}^{\mathrm{NMT}}$ $, \frac{{{(D}_{0}-\alpha c_{1})}^{2}\alpha\eta^{2}}{\left( 3\alpha\eta-\gamma^{2} \right)^{2}}\geq\frac{{{(D}_{0}-\alpha c_{1})}^{2}\alpha\eta^{2}}{{(4\alpha\eta-\gamma^{2})}^{2}}$is true. From$\text{p}_{\text{1}}^{\text{NM}}\leq\text{p}_{\text{1}}^{\text{NMT}},\frac{{2D}_{0}\eta+\left( \alpha\eta-\gamma^{2} \right)c_{1}}{3\alpha\eta-\gamma^{2}}\leq\frac{{3\eta D}_{0}+\left( \alpha\eta-\gamma^{2} \right)c_{1}}{4\alpha\eta-\gamma^{2}},$ simplified to ${(D}_{0}-\alpha c_{1}) \left( \alpha\eta-\gamma^{2} \right)\geq0,$then when $\alpha\eta-\gamma^{2}\geq0$,the inequality is true.

Then, Theorem 10 is proven.

**Proof of Theorem 11.**

Because $D^{\mathrm{NR}}=D^{\mathrm{NM}}$，$D^{\mathrm{NRT}}=D^{\mathrm{NMT}}$，$s_{m}^{\mathrm{NR}}=s_{m}^{\mathrm{NM}}$，$s_{t}^{\mathrm{NRT}}=s_{t}^{\mathrm{NMT}}$，$\pi_{m}^{\mathrm{NR}}=\pi_{r}^{\mathrm{NM}}$，$\pi_{m}^{\mathrm{NRT}}=\pi_{r}^{\mathrm{NMT}}$，$\pi_{r}^{\mathrm{NR}}=\pi_{m}^{\mathrm{NM}}$，$\pi_{r}^{\mathrm{NRT}}=\pi_{m}^{\mathrm{NMT}}$，$\text{p}_{\text{1}}^{\text{NR}}=\text{p}_{\text{1}}^{\text{NM}}$,$\text{p}_{\text{1}}^{\text{NRT}}=\text{p}_{\text{1}}^{\text{NMT}}$, it can be proved in the same way as Theorem 10.

Then, Theorem 11 is proven.

**Proof of Theorem 12.**

When manufacturers conduct green product R&D, $\mathrm{From}D^{\mathrm{NM}}\geq D^{\mathrm{RM}}\geq D^{\mathrm{MM}},we can obtain \frac{{(D}_{0}-\alpha c_{1})\alpha\eta}{3\alpha\eta-\gamma^{2}}\geq\frac{\left( {(D}_{0}-\alpha c_{1} \right)\alpha\eta}{\text{2(2}\alpha\eta-\gamma^{2})}\geq\frac{{(D}_{0}-\alpha c_{1})\alpha\eta}{4\alpha\eta-\gamma^{2}}. By$simplifying $\text{2(2}\alpha\eta-\gamma^{2})\geq3\alpha\eta-\gamma^{2},$we can get $\alpha\eta-\gamma^{2}\geq0$.When retailers conduct green product R&D, the same can be proved because $\text{D}^{\mathrm{MR}}=\text{D}^{\text{RM}}$,$D^{\mathrm{RR}}=D^{\mathrm{MM}}$,$D^{\mathrm{NR}}=D^{\mathrm{NM}}$. When manufacturers and retailers jointly conduct green product R&D, from $D^{\mathrm{NC}}\geq D^{\mathrm{MC}}=D^{\mathrm{RC}}$,$we can obtain \text{4}\alpha\eta-3\gamma^{2}\geq3\alpha\eta-2\gamma^{2}$,when $\alpha\eta-\gamma^{2}\geq0,$the inequality is true. When manufacturers outsource green product R&D to third-party companies, from $D^{\mathrm{NMT}}\geq D^{\mathrm{RMT}}=D^{\mathrm{MMT}}$,we can obtain $\frac{{(D}_{0}-\alpha c_{1})\alpha\eta}{4\alpha\eta-\gamma^{2}}\geq\frac{{(D}_{0}-\alpha c_{1})\alpha\eta}{2(3\alpha\eta-\gamma^{2})}$, simplified to $2\alpha\eta-\gamma^{2}\geq0$, the inequality is true. When retailers outsource green product R&D to third-party companies, because $D^{\mathrm{NRT}}=D^{\mathrm{NMT}}$,$D^{\mathrm{RMT}}=D^{\mathrm{MMT}}=D^{\mathrm{RRT}}=D^{\mathrm{MRT}}$,$D^{\mathrm{NRT}}\geq D^{\mathrm{RRT}}=D^{\mathrm{MRT}}$ is true.

Then, Theorem 12 is proven.

**Proof of Theorem 13.**

When manufacturers conduct green product R&D, from $s_{m}^{\mathrm{NM}}\geq s_{m}^{\mathrm{RM}}\geq s_{m}^{\mathrm{MM}}$,$we can obtain \frac{{(D}_{0}-\alpha c_{1})\gamma}{3\alpha\eta-\gamma^{2}}\geq\frac{\left( D_{0}-\alpha c_{1} \right)\gamma}{2\left( 2\alpha\eta-\gamma^{2} \right)}\geq\frac{{(D}_{0}-\alpha c_{1})\gamma}{4\alpha\eta-\gamma^{2}}$. By simplifying $\text{2(2}\alpha\eta-\gamma^{2})\geq3\alpha\eta-\gamma^{2}$, we can get $\alpha\eta-\gamma^{2}\geq0$.When retailers conduct green product R&D, the same can be proved because $s_{r}^{\mathrm{MR}}=s_{m}^{\mathrm{RM}}$, $s_{r}^{\mathrm{RR}}=s_{m}^{\mathrm{MM}}$, $s_{r}^{\mathrm{NR}}=s_{m}^{\mathrm{NM}}$, then $s_{r}^{\mathrm{NR}}\geq s_{r}^{\mathrm{MR}}\geq s_{r}^{\mathrm{RR}}$is true. When manufacturers and retailers conduct jointly green products R&D, from $s_{m}^{\mathrm{NC}}\geq s_{m}^{\mathrm{MC}}=s_{m}^{\mathrm{RC}}$,$we can obtain \frac{\left( {(D}_{0}-\alpha c_{1} \right)\gamma}{\text{4}\alpha\eta-3\gamma^{2}}\geq\frac{\left( {(D}_{0}-\alpha c_{1} \right)\gamma}{3\alpha\eta-2\gamma^{2}}$. By simplifying $4\alpha\eta-3\gamma^{2}\geq3\alpha\eta-2\gamma^{2}$, we can get $\alpha\eta-\gamma^{2}\geq0$. When manufacturers outsource green product R&D to third-party companies, from$s_{t}^{\mathrm{NMT}}\geq s_{t}^{\mathrm{MMT}}=s_{t}^{\mathrm{RMT}}$,$\frac{{(D}_{0}-\alpha c_{1})\gamma}{4\alpha\eta-\gamma^{2}}\geq\frac{{(D}_{0}-\alpha c_{1})\gamma}{2(3\alpha\eta-\gamma^{2})}$, simplified to$2\alpha\eta-\gamma^{2}\geq0$. When retailers outsource green product R&D to third-party companies, because $s_{t}^{\mathrm{NRT}}=s_{t}^{\mathrm{NMT}}$,$s_{t}^{\mathrm{MMT}}=s_{t}^{\mathrm{RMT}}=s_{t}^{\mathrm{RRT}}=s_{t}^{\mathrm{MRT}}, s_{t}^{\mathrm{NRT}}\geq s_{t}^{\mathrm{RRT}}=s_{t}^{\mathrm{MRT}}$ is true.

Then, Theorem 13 is proven.

**Proof of Theorem 14.**

When manufacturers conduct green product R&D, from $p_{1}^{\mathrm{MM}}\geq\text{p}_{\text{1}}^{\text{RM}}$,we can obtain$\frac{3D_{0}\eta+\left( \alpha\eta-\gamma^{2} \right)c_{1}}{4\alpha\eta-\gamma^{2}}\geq\frac{\text{(3}\alpha\eta-\gamma^{2}\text{)}{(D}_{0}\text{+(}\alpha\eta-\gamma^{2}\text{)}\alpha c_{1}}{\text{2α(2}\alpha\eta-\gamma^{2})}$. By simplifying ${(D}_{0}-\alpha c_{1})(\alpha\eta-\gamma^{2}) \geq0$, we can get $\alpha\eta-\gamma^{2}\geq0$. From $\text{p}_{\text{1}}^{\text{RM}}\geq p_{1}^{\mathrm{NM}}$,we can obtain$\frac{\text{(3}\alpha\eta-\gamma^{2}\text{)}{(D}_{0}\text{+(}\alpha\eta-\gamma^{2}\text{)}\alpha c_{1}}{\text{2α(2}\alpha\eta-\gamma^{2})}\geq\frac{{2D}_{0}\eta+\left( \alpha\eta-\gamma^{2} \right)c_{1}}{3\alpha\eta-\gamma^{2}}$, simplified to$D_{0}-\alpha c_{1}\geq0$. When retailers conduct green product R&D, the same can be proved because $p_{1}^{\mathrm{MM}}=p_{1}^{\mathrm{RR}},\text{p}_{\text{1}}^{\text{RM}}=\text{p}_{\text{1}}^{\text{MR}}$,$p_{1}^{\mathrm{NM}}=p_{1}^{\mathrm{NR}}$,$p_{1}^{\mathrm{RR}}\geq\text{p}_{\text{1}}^{\text{MR}}\geq p_{1}^{\mathrm{NR}}$ is true. When manufacturers outsource green product R&D to third-party companies, from $p_{1}^{\mathrm{MMT}}=p_{1}^{\mathrm{RMT}}\geq p_{1}^{\mathrm{NMT}}$,$\frac{\left( 5\alpha\eta-\gamma^{2} \right)D_{0}+\left( \alpha\eta-\gamma^{2} \right)\alpha c_{1}}{2\alpha(3\alpha\eta-\gamma^{2})}\geq\frac{{3\eta D}_{0}+(\alpha\eta-\gamma^{2})c_{1}}{4\alpha\eta-\gamma^{2}}$, simplified to$D_{0}-\alpha c_{1}\geq0$. When retailers outsource green product R&D to third-party companies, because $p_{1}^{\mathrm{MRT}}=p_{1}^{\mathrm{MMT}}$，$p_{1}^{\mathrm{RRT}}=p_{1}^{\mathrm{RMT}}$，$p_{1}^{\mathrm{NRT}}=p_{1}^{\mathrm{NMT}},p_{1}^{\mathrm{MRT}}=p_{1}^{\mathrm{RRT}}\geq p_{1}^{\mathrm{NRT}}$ is true.

Then, Theorem 14 is proven.

**Proof of Theorem 15.**

When manufacturers conduct green product R&D, from $\pi_{m}^{\mathrm{MM}}\geq\pi_{m}^{\mathrm{NM}}, by$the simplificationof$\frac{{{(D}_{0}-\alpha c_{1})}^{2}\eta}{2\left( 4\alpha\eta-\gamma^{2} \right)}\geq\frac{{{(D}_{0}-\alpha c_{1})}^{2}(2\alpha\eta-\gamma^{2})\eta}{2\left( 3\alpha\eta-\gamma^{2} \right)^{2}}$, $\alpha^{2}\eta^{2}\geq0$ is true. From $\pi_{m}^{\mathrm{NM}}\geq\pi_{m}^{\mathrm{RM}}$,$\frac{{{(D}_{0}-\alpha c_{1})}^{2}(2\alpha\eta-\gamma^{2})\eta}{2\left( 3\alpha\eta-\gamma^{2} \right)^{2}}\geq\frac{{{(D}_{0}-\alpha c_{1})}^{2}\eta}{8\left( 2\alpha\eta-\gamma^{2} \right)}$,simplified to ${7\alpha}^{2}\eta^{2}-10\alpha\eta\gamma^{2}+3\gamma^{4}=(\alpha\eta-\gamma^{2})(7\alpha\eta-3\gamma^{2})\geq0$,then$\alpha\eta-\gamma^{2}\geq0$. From $\pi_{r}^{\mathrm{RM}}\geq\pi_{r}^{\mathrm{NM}},$the simplification$\mathrm{of}\frac{\left( D_{0}-\alpha c_{1} \right)^{2}\eta}{\text{4(2}\alpha\eta-\gamma^{2})}\geq\frac{{{(D}_{0}-\alpha c_{1})}^{2}\alpha\eta^{2}}{\left( 3\alpha\eta-\gamma^{2} \right)^{2}}$, ${(\alpha\eta-\gamma^{2})}^{2}\geq0$ is true. From $\pi_{r}^{\mathrm{NM}}\geq\pi_{r}^{\mathrm{MM}}$,$the simplification of \frac{{{(D}_{0}-\alpha c_{1})}^{2}\alpha\eta^{2}}{\left( 3\alpha\eta-\gamma^{2} \right)^{2}}\geq\frac{{{(D}_{0}-\alpha c_{1})}^{2}\alpha\eta^{2}}{\left( 4\alpha\eta-\gamma^{2} \right)^{2}}$,$7\alpha\eta-2\gamma^{2}\geq0$ is true.

When retailers conduct green product R&D, the same can be proved because $\pi_{m}^{\mathrm{MR}}=\pi_{r}^{\mathrm{RM}}$, $\pi_{m}^{\mathrm{RR}}=\pi_{r}^{\mathrm{MM}}$, $\pi_{m}^{\mathrm{NR}}=\pi_{r}^{\mathrm{NM}}, \pi_{r}^{\mathrm{RR}}=\pi_{m}^{\mathrm{MM}}, \pi_{r}^{\mathrm{NR}}=\pi_{m}^{\mathrm{NM}}, \pi_{r}^{\mathrm{MR}}=\pi_{m}^{\mathrm{RM}}$,$\pi_{m}^{\mathrm{MR}}\geq\pi_{m}^{\mathrm{NR}}\geq\pi_{m}^{\mathrm{RR}}$,$\pi_{r}^{\mathrm{RR}}\geq\pi_{r}^{\mathrm{NR}}\geq\pi_{r}^{\mathrm{MR}}$ is true.

When manufacturers and retailers jointly conduct green product R&D, from $\pi_{m}^{\mathrm{MC}}\geq\pi_{m}^{\mathrm{NC}},$by the simplification of $\frac{{{(D}_{0}-\alpha c_{1})}^{2}\eta}{\text{2(4}\alpha\eta-3\gamma^{2})}\geq\frac{{{(D}_{0}-\alpha c_{1})}^{2}\left( 2\alpha\eta-\gamma^{2} \right)\eta}{2\left( 3\alpha\eta-2\gamma^{2} \right)^{2}}, {(\alpha\eta-\gamma^{2})}^{2}\geq0$ is true. From ${\pi_{m}^{\mathrm{NC}}\geq\pi}_{m}^{\mathrm{RC}},$by the simplification of $\frac{{{(D}_{0}-\alpha c_{1})}^{2}(2\alpha\eta-\gamma^{2})\eta}{2\left( 3\alpha\eta-2\gamma^{2} \right)^{2}}\geq\frac{{{(D}_{0}-\alpha c_{1})}^{2}(2\alpha\eta-\gamma^{2})\eta}{\text{2}{\text{(4}\alpha\eta-3\gamma^{2})}^{2}}, \alpha\eta-\gamma^{2}\geq0$ is true. Because $\pi_{r}^{\mathrm{RC}}=\pi_{m}^{\mathrm{MC}},\pi_{r}^{\mathrm{NC}}=\pi_{m}^{\mathrm{NC}},\pi_{r}^{\mathrm{MC}}=\pi_{m}^{\mathrm{RC}},$then $\pi_{r}^{\mathrm{RC}}\geq\pi_{r}^{\mathrm{NC}}\geq\pi_{r}^{\mathrm{MC}}$ is true.

When manufacturers outsource green product R&D to third-party companies, from $\pi_{m}^{\mathrm{MMT}}\geq\pi_{m}^{\mathrm{NMT}}$,$\frac{{{(D}_{0}-\alpha c_{1})}^{2}\eta}{4(3\alpha\eta-\gamma^{2})}\geq\frac{{{(D}_{0}-\alpha c_{1})}^{2}\alpha\eta^{2}}{{(4\alpha\eta-\gamma^{2})}^{2}}$, simplified to ${(2\alpha\eta-\gamma^{2})}^{2}\geq0$. From $\pi_{m}^{\mathrm{NMT}}\geq\pi_{m}^{\mathrm{RMT}},$ $\frac{{{(D}_{0}-\alpha c_{1})}^{2}\alpha\eta^{2}}{{(4\alpha\eta-\gamma^{2})}^{2}}\geq\frac{{{(D}_{0}-\alpha c_{1})}^{2}\alpha\eta^{2}}{4{(3\alpha\eta-\gamma^{2})}^{2}}$, simplified to ${(2\alpha\eta-\gamma^{2})}^{2}\geq0$.Because$\pi_{m}^{\mathrm{MMT}}=\pi_{r}^{\mathrm{RMT}}$，$\pi_{m}^{\mathrm{NMT}}=\pi_{r}^{\mathrm{NMT}}$，$\pi_{m}^{\mathrm{RMT}}=\pi_{r}^{\mathrm{MMT}}$,$\pi_{r}^{\mathrm{RMT}}\geq\pi_{r}^{\mathrm{NMT}}\geq\pi_{r}^{\mathrm{MMT}}$is true.

When retailers outsource green product R&D to third-party companies, because$\pi_{m}^{\mathrm{MMT}}=\pi_{m}^{\mathrm{MRT}}$,$\pi_{m}^{\mathrm{RMT}}=\pi_{m}^{\mathrm{RRT}}$,$\pi_{m}^{\mathrm{NMT}}=\pi_{m}^{\mathrm{NRT}}$,$\pi_{r}^{\mathrm{RMT}}=\pi_{r}^{\mathrm{RRT}}$,$\pi_{r}^{\mathrm{NMT}}=\pi_{r}^{\mathrm{NRT}}$,$\pi_{r}^{\mathrm{MMT}}=\pi_{r}^{\mathrm{MRT}}$,when $2\alpha\eta-\gamma^{2}\geq0$,$\pi_{m}^{\mathrm{MRT}}\geq\pi_{m}^{\mathrm{NRT}}\geq\pi_{m}^{\mathrm{RRT}}$ and $\pi_{r}^{\mathrm{RRT}}\geq\pi_{r}^{\mathrm{NRT}}\geq\pi_{r}^{\mathrm{MRT}}$ are true.

Then, Theorem 15 is proven.
